# The influence of the crystal sponge framework on guest molecule conformation†

**DOI:** 10.1039/d4ce00699b

**Published:** 2025-01-31

**Authors:** Eleanor M. Soper, Simon J. Coles, Graeme M. Day

**Affiliations:** a School of Chemistry, University of Southampton Southampton SO17 1BJ UK e.soper@soton.ac.uk s.j.coles@soton.ac.uk g.m.day@soton.ac.uk

## Abstract

The crystalline sponge (CS) method has become an important technique for structural elucidation of compounds that are challenging to crystallise. The impact of the CS environment on guest molecule conformations has not been systematically studied. We present a computational investigation of the conformations of organic molecules of varying flexibility in a set of experimentally determined CS structures, comparing them to gas phase conformers and, where available, pure and co-crystal structures. *Via* solid state and molecular density functional theory calculations, we quantify the total relative energy, conformational energy, and intramolecular strain of guest molecules, as well as framework strain. Our results show that while CS structures induce distortion in guest geometries (total relative energies up to 41 kJ mol^−1^), they generally adopt low-energy conformations, often within 2 kJ mol^−1^ of the global energy minimum. Intramolecular strain in CS structures is often lower than in conventional crystal structures, suggesting a more neutral packing environment where molecules are closer to their favoured isolated-molecule geometries. We also observe that multiple guests can influence each other's geometries, even in the absence of direct guest–guest interactions. These findings provide a quantification of conformational distortion that can form the basis for interpreting molecular geometries obtained from CS structures.

## Abstract 2

This study provides fundamental understanding of the impact of the crystalline sponge on guest molecule conformation by evaluating geometric distortion from ideal, gas phase calculated geometries and the related energetic cost.

## Graphical abstract

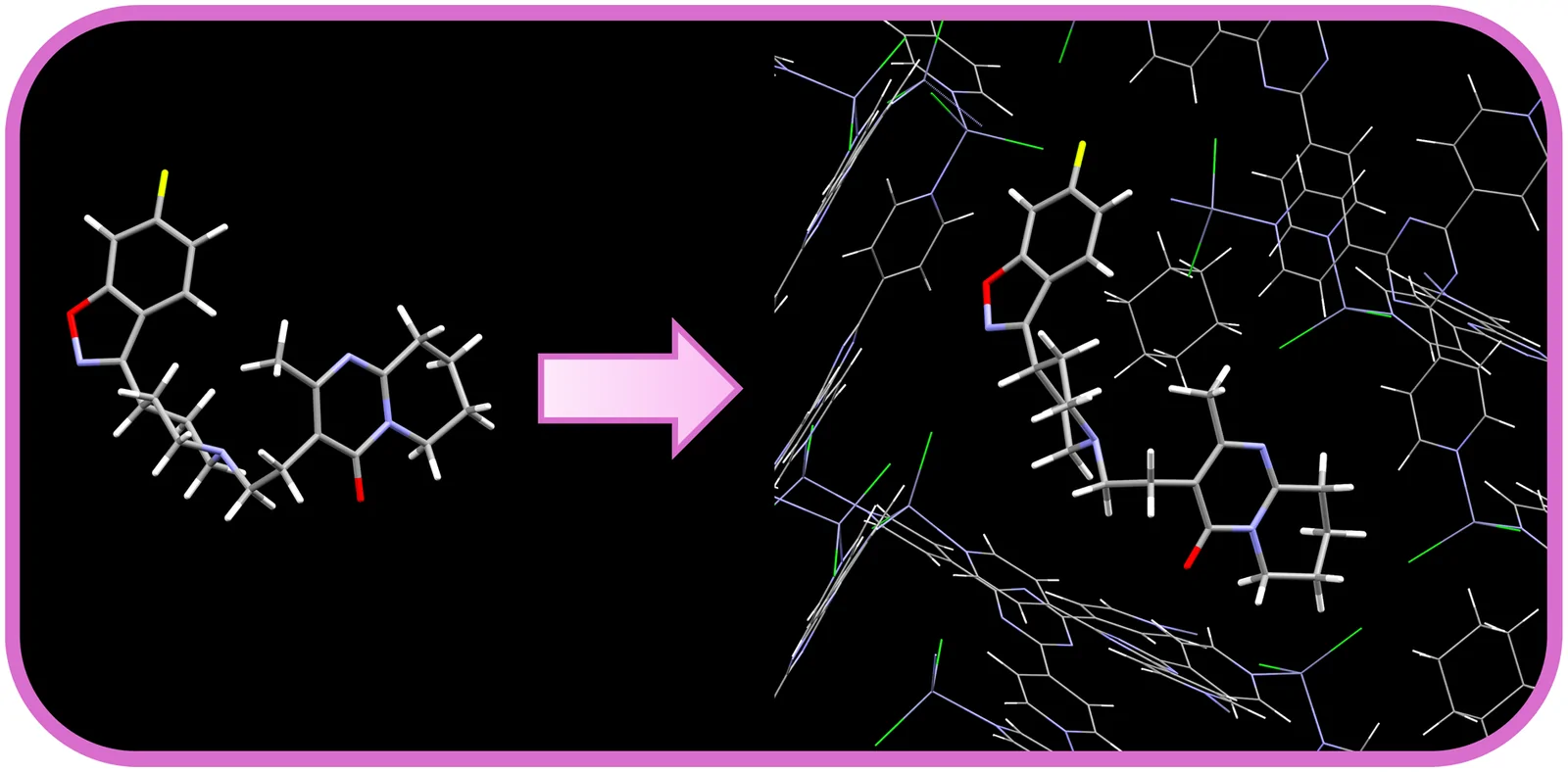

## 1 Introduction

The accurate determination of molecular structure is of critical importance in chemical research. Single crystal X-ray diffraction (SCXRD) is considered to be one of the most powerful methods of structure elucidation, giving the ability to determine the absolute configuration and stereochemistry of molecules with relative ease. A major drawback of the technique is that, as the name suggests, a good-quality single crystal needs to be produced. Many synthetic organic or natural products can be difficult to crystallise, produced in minute quantities, or are liquid at ambient temperatures, and as a result are often unsuitable for SCXRD analysis.

The crystal sponge (CS) method, first reported by Fujita and coworkers^1^ in 2013, has become an important technique in the structural elucidation of compounds considered “uncrystallisable”. The technique removes the necessity of sample crystallisation by utilising the porosity of crystalline metal–organic frameworks (MOFs) to form host–guest complexes with the desired compound, which can then be analysed by SCXRD.^2^ The CS method is particularly useful in pharmaceutical research, where knowing the exact structure and stereochemical information of a molecule is of critical importance.

The most successful and generally applicable MOF used for the CS method is the [((ZnX_2_)_3_(tpt)_2_·*x*(solvent))_*n*_] system, (tpt = 2,4,6-tris(4-pyridyl)-1,3,5-triazine ligand; X = I, Br, Cl). The system has several advantageous properties including large, hydrophobic pores (5 × 8 Å) with electron-deficient binding sites offered by the tpt ligand. The ZnX_2_ components and pyridyl protons of the tpt ligands provide hydrogen-bond donor and acceptor sites, respectively, which enables reproducible and long-range ordering of the guests within the MOF pores and absorption of organic and aromatic molecules of a range of sizes. The interpenetrated framework allows for some flexibility and expansion of the pore size to accommodate larger guests.^3,4^

The study of MOFs and host–guest complexes by computational methods is a growing area of research, dominated by studies aiming to model the uptake of gases, drug delivery, and calculation of binding energies and interaction strengths of small molecules in the pores of frameworks by molecular dynamics, Monte Carlo simulations, and DFT methods.^5–9^ There have been fewer studies directed at MOFs containing flexible molecules, and specifically CS structures. There are certain challenges that arise due to the size and complexity of these systems, especially when considering the presence of large guest molecules and solvent(s); however, the development of molecular simulation methods and access to more powerful high performance computing has meant that modelling such systems is more accessible, and has led to an increasing number of MOFs being studied computationally.

While there are many examples of computational approaches being applied to MOFs, the (ZnX_2_)_3_(tpt)_2_ structures most commonly used for the CS method are not well-studied from a computational perspective, despite the large subset of >500 CS structures available in the Cambridge Structural Database^10^ (CSD). Investigation into the CS method has primarily focused on the determination of novel, otherwise intractable, molecular structures and the improvement of experimental procedures.^3,11–16^ At the time of writing, the only study of CS structures using computational methods that the authors are aware of is the work of Cardenal and Ramadhar,^17^ who demonstrated the use of dispersion-corrected density functional theory (DFT) to analyse a set of previously-published experimental crystalline sponge structures and obtained binding energies of guests within the pores of the framework.

This study seeks to provide a fundamental understanding of the extent to which the CS structure impacts the geometry of guest molecules and the type of environment that is present in the pores of the CS. The geometries of guest molecule(s) from the optimised CS structures are compared to the DFT conformer landscape to quantify the intramolecular strain and conformational energetics of the distortion induced by the pore environment. Comparison to pure and cocrystal structures, where available, aids further understanding of the exact nature of the pore, defining if the environment of the CS is similar or different from typical, purely organic crystal structures.

## 2 Methods

### 2.1 Geometry optimisations

The first step is to obtain geometric and energetic information for the conformations of a molecule in its observed crystal structures. The observed molecular geometries and energies are evaluated after optimisation of the experimentally determined crystal structures, which removes any experimental artefacts and errors in atomic positions remaining after structure refinement.

For consistency, the same level of theory and calculation settings are used for all crystal structure and single molecule energy calculations. All density functional theory (DFT) calculations were performed in the CRYSTAL17 (ref. 18) software using the POB-TZVP basis set^19,20^ and B3LYP functional^21^ in combination with the D3 dispersion correction.^22^ To account for the basis set superposition error (BSSE) that arises in finite basis sets, the geometric counterpoise (gCP) correction^23^ was implemented in all calculations.

**Fig. 1:**
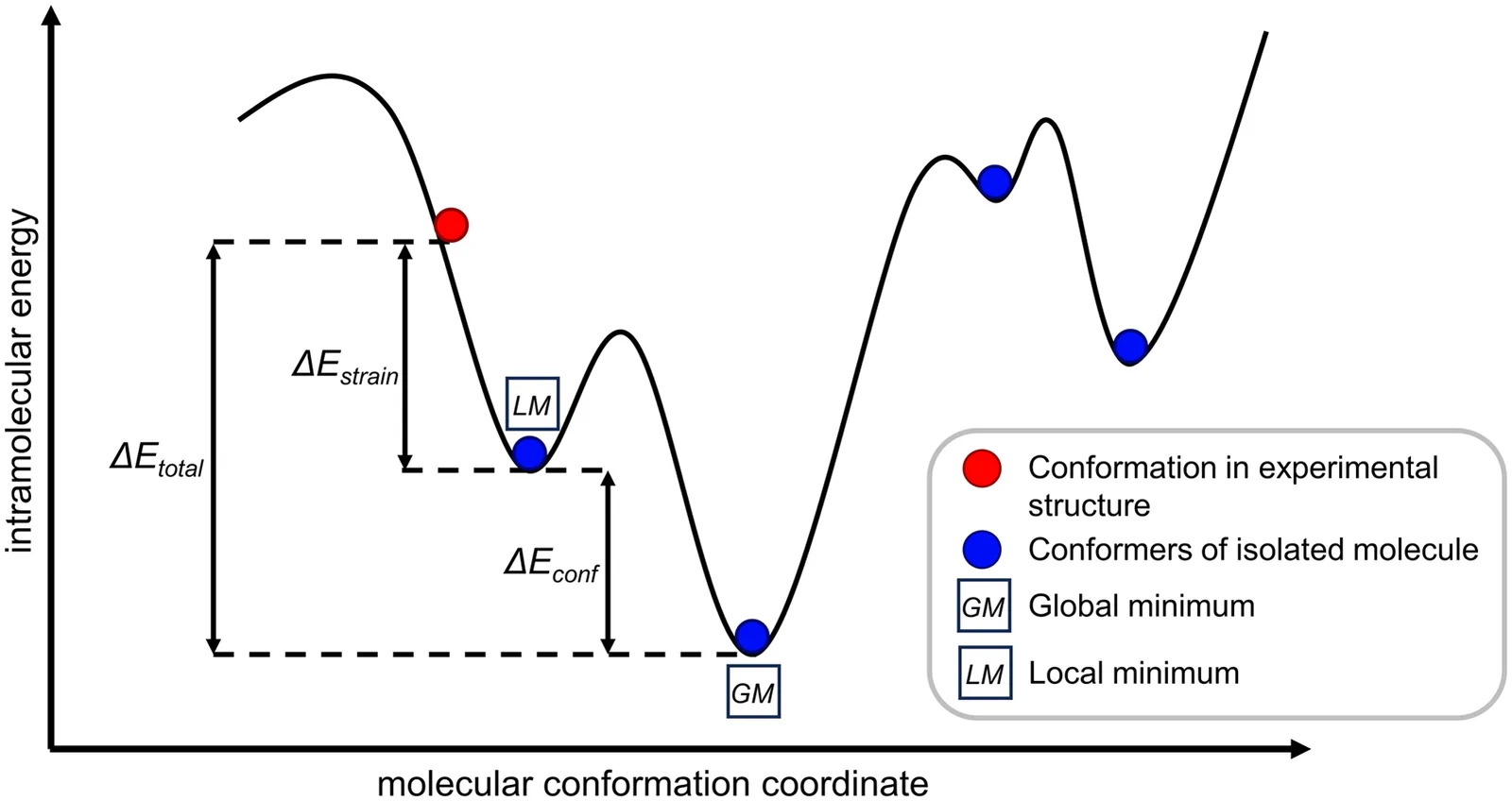
Schematic of a conformational energy surface: the molecular energy *vs.* a general conformational degree of freedom. The molecular geometry found in the experimental crystal structure is represented as a red point and the conformers of the isolated molecule are represented as blue points. The global minimum conformer, at the lowest point on the potential energy landscape, is indicated by GM. The local minimum conformer of the guest in the experimental structure is indicated by LM.

The symmetry-independent (unique) guest molecules were extracted from the DFT optimised experimental crystal structures. Single-point energy calculations were performed to obtain the energies of the isolated molecules from their crystalline state, *E*^mol^_crystal_ (see red point in Fig. 1). The crystalline molecular geometry was also used to obtain the associated local energy minimum on the conformational energy surface, *E*^opt,mol^_local_, by running unconstrained geometry optimisations to relax the molecule to the nearest local energy minimum (blue point marked LM in Fig. 1). The energy difference between the single-point and relaxed molecular geometries of the unique molecules within the crystal structures is the intramolecular strain energy, Δ*E*_strain_ (see Fig. 1 and eqn (1)):

1 Δ*E*_strain_ = *E*^mol^_crystal_ − *E*^opt,mol^_local_

which is an energetic measure of the distortion of the molecule in the CS away from its nearest stable gas phase geometry.

### 2.2 Conformational searches

The CREST conformer search^24^ method was applied to generate complete sets of conformers for each guest molecule studied. To ensure thorough sampling, we generated multiple CREST searches from diverse starting structures to fully search the conformational landscape. Duplicates from the combined searches are removed by clustering based on torsion angles within a maximum energy window of 2 kJ mol^−1^ and torsion angle tolerances were set to 10.0° and 5.0° for the single angle maximum and the maximum RMS angle, respectively.

All unique conformers resulting from the initial search were re-optimised with DFT-D3, applying the same computational parameters as described in section 2.1. The result should be a complete set of low energy conformers for each molecule. The DFT-optimised conformers were re-clustered to remove duplicates, resulting in the final DFT conformer landscape.

To identify the local conformer of the isolated molecule from the observed crystal structures in the conformational landscape, geometric comparisons with the sets of conformers were performed. The difference in energy between the lowest energy generated conformer (the global energy minimum conformer), *E*_GM_, and the energy of the local conformer corresponding to the crystalline molecular geometry, *E*^opt,mol^_local_, is referred to as the conformational energy, Δ*E*_conf_ (see Fig. 1 and eqn (2)):

2 Δ*E*_conf_ = *E*^opt,mol^_local_ − *E*_GM_

As such, once the energy of the global energy minimum conformer is known, the total relative energy of the isolated molecule from the experimental crystal structure, Δ*E*_total_, can be found as described in eqn (3):

3 Δ*E*_total_ = Δ*E*_strain_ + Δ*E*_conf_ = *E*^mol^_crystal_ − *E*_GM_

which is the total energy difference between the molecular conformation in the optimised CS structure and the most stable gas phase molecular geometry. Δ*E*_total_, and its partitioning into Δ*E*_strain_ and Δ*E*_conf_, quantify the influence, in energetic terms, of the CS environment on the guest molecule geometry.

### 2.3 Framework strain

The strain energy of the MOF was also quantified to provide insight into the distortion of the framework that occurs on uptake of the guest molecule. All guest and solvent molecules were removed from the optimised CS structure, leaving the empty (ZnX_2_)_3_(tpt)_2_ framework. The energy of the MOF in the optimised CS structure *E*^crystal^_MOF_ was calculated by running a single point energy calculation of the empty framework. An unconstrained geometry optimisation was also performed on the empty MOF structure, applying the same calculation settings as described above. The energy difference between the single-point and relaxed empty MOF structures (*E*^opt,MOF^_local_) is the intramolecular strain energy of the framework, Δ*E*_MOF_ (eqn (4)):

4 Δ*E*_MOF_ = *E*^MOF^_crystal_ − *E*^opt,MOF^_local_

Since the space group varies between structures, framework strain energies are quoted per minimum framework unit (ZnX_2_)_3_(tpt)_2_, or 81 atoms, to maintain consistency between structures.

### 2.4 Selection criteria for crystalline sponge structures

In order to gain insights into the effect of the CS framework on the conformation of guest molecules within the pore, it is important to choose a set of experimental structures that reflect the range of flexibility of guests that have been soaked into CS. While this set must be representative of the full range of different types of analytes, it must also comprise of experimental structures that are of a suitable quality to be a starting point for computational analysis. Making decisions solely based on individual quantitative crystallographic statistics, such as the *R*-factor, cannot be relied upon as there are many factors, including some qualitative ones, which contribute to the completeness of a CS structure. Therefore, the selection process requires manual filtering to sort through the CS candidates and produce a subset for study. The considerations that have been used in this process, and their justification, are provided in the ESI.†

To identify CS structures for this study, the CSD was searched based on the ZnX_2_ (X = Cl, Br, I) and tpt ligand features of the (ZnX_2_)_3_(tpt)_2_ framework. The subset of CS structures were chosen from the resulting CSD hits and selected based on the following criteria: (1) the guest molecule satisfies Lipinski's rule of five, (2) no solvent masking methods have been used in the structure refinement, and (3) there is minimal residual solvent accessible void space. The number of rotatable bonds, excluding methyl groups, was also considered to ensure the set of molecules included the full range of possible guest flexibility.

### 2.5 Preparation of the dataset

CS structures, *i.e.* those with isolated molecules residing in framework pores, typically have more disorder than conventional organic crystal structures. This can be present in both the framework and also the guest molecules in the pore, which can comprise both the analyte of interest and solvent molecules. The guest molecules can be situated over a symmetry site, or due to low occupancy *i.e.* not being present in exactly the same location in every pore, found in multiple positions, orientations, or disordered with solvent. Consequently, it is important to consider how disorder affects experimental structures on a case-by-case basis to ensure that the structure taken forward for computational study is as realistic as possible. Full details for dealing with framework, guest and solvent disorder is described in the ESI.†

### 2.6 Structures chosen for study

**Fig. 2:**
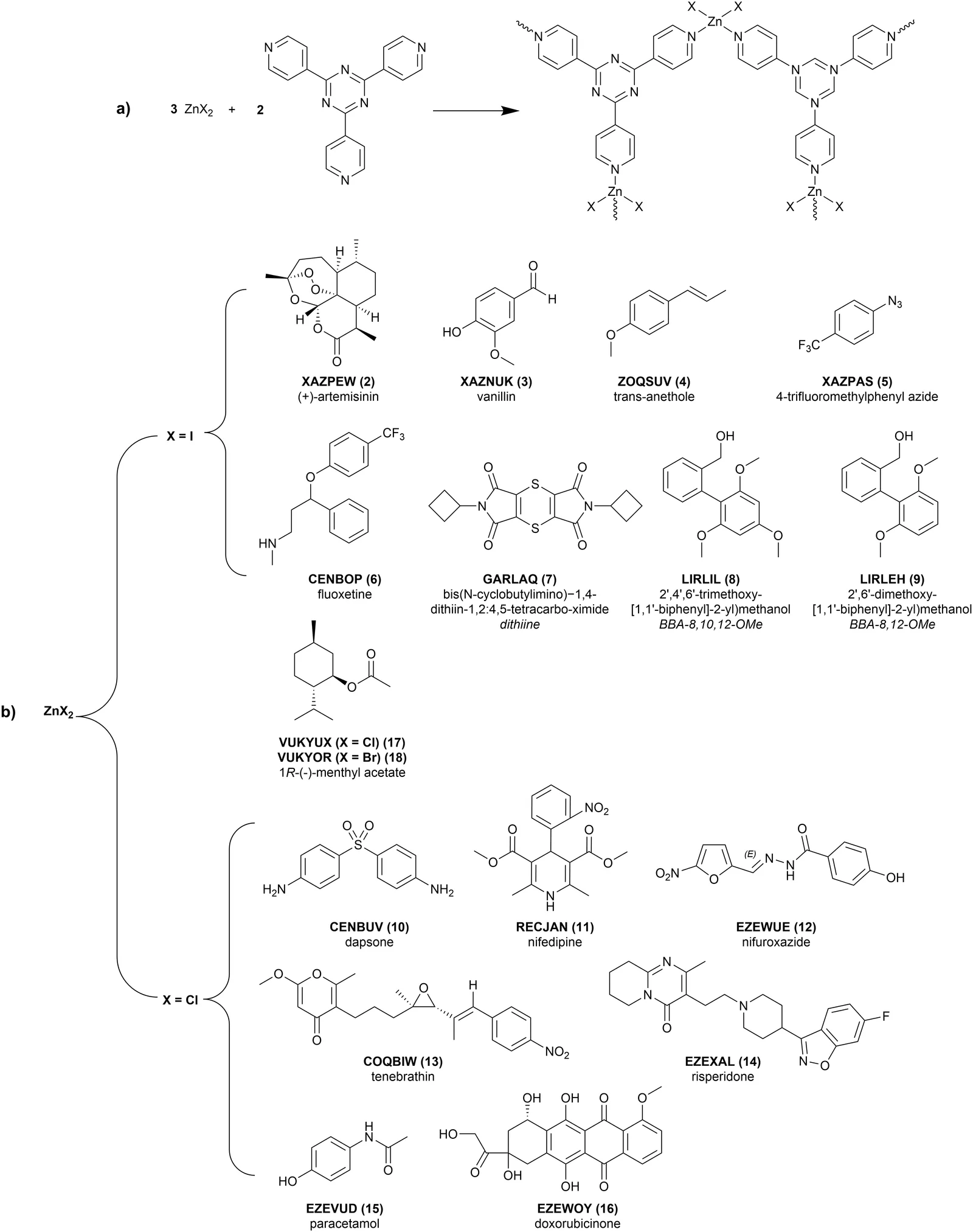
(a) Schematic for the synthesis of the (ZnX_2_)_3_(tpt)_2_ crystalline sponges, where X = Cl, Br, or I. The zinc(ii) ions coordinate to the tpt ligands to form the displayed repeat unit. (b) Chemical diagrams of the guest molecules and associated CSD reference codes of the studied crystalline sponge structures. Each structure includes the guest molecule soaked into the (ZnX_2_)_3_(tpt)_2_ framework.

Beyond filtering on quality, the 17 CS structures used in this study were selected primarily to reflect the range of conformation flexibility of guest molecules that have successfully been soaked into this CS framework. The 16 unique guests are detailed in Fig. 2. The flexibility distribution, based on the number of rotatable bonds (excluding methyl groups), can be found in the ESI.† An additional, guest-free (solvent-only), structure (1, LABMOT) was also included in the structure set for the purpose of framework strain analysis. If available, the single-component (pure) crystal structures and any co-crystal and/or solvate structures of the guest molecules were also studied to provide comparison.

## 3 Results and discussion

As many of the chosen structures contain some guest and/or solvent disorder, multiple configurations have been optimised to consider any effects of positional changes in the pores of the CS. The resulting values of Δ*E*_strain_ and Δ*E*_conf_ vary by <1 kJ mol^−1^ between configurations; this small energy difference is consistent with the presence of disorder. For discussion, in all further analysis we only include the CS structure configurations with the lowest energy of the guest molecule. These results are summarised in Table 1. The full results are reported in Table S2.†

**Table 1:** Calculated strain, conformational, and total relative energies of the observed molecular geometries and RMSD of atomic positions of the guest with its optimised isolated conformer. Only the structure configurations with the lowest energies are reported here. Where multiple guests are present, these are labelled A to D

MOF type		CSD Refcode	Config.^a^	Guest	Δ*E*_strain_ (kJ mol^−1^)	RMSD (Å)	Δ*E*_conf_ (kJ mol^−1^)	Δ*E*_total_ (kJ mol^−1^)	Δ*E*_MOF_^b^ (kJ mol^−1^)
ZnI_2_	1	LABMOT^3^	1		—	—	—	—	16.06
	2	XAZPEW^14^	1		3.99	0.042	0.00	3.99	4.53
	3	XAZNUK^14^	1	A	9.87	0.060	4.24	14.10	19.83
				B	3.31	0.071	0.01^c^	3.33	
				C	2.41	0.034	0.01^c^	2.42	
				D	7.81	0.124	0.01^c^	7.81	
	4	ZOQSUV^12^	1	A	4.09	0.127	0.17	4.26	8.79
				B	3.37	0.129	0.16	3.53	
				C	6.73	0.101	0.18	6.90	
	5	XAZPAS^14^	1		2.50	0.094	0.00	2.50	7.19
	6	CENBOP^25^	1		17.79	0.463	8.74	26.53	8.49
	7	GARLAQ^26^	1		18.16	0.185	0.00	18.16	16.82
	8	LIRLIL^27^	2	A	23.39	0.577	5.13	28.52	9.98
				B	6.75	0.102	21.78	28.53	
	9	LIRLEH^27^	1	A	19.93	0.423	3.81	23.76	9.10
				B	5.23	0.085	21.05	26.28	
ZnCl_2_	10	CENBUV^25^	3		13.52	0.384	1.79	15.31	100.95
	11	RECJAN^25^	1		25.91	0.651	6.74	32.65	16.89
	12	EZEWUE^28^	3		10.99	0.385	0.57	11.56	14.26
	13	COQBIW^29^	1	A	12.56	0.404	28.45	41.01	17.49
				B	16.24	0.481	19.98	36.22	
	14	EZEXAL^28^	6	A	12.46	0.432	6.82	19.28	18.07
				B	18.39	0.965	5.03	23.42	
	15	EZEVUD^28^	1		6.90	0.112	1.59	8.50	13.02
	16	EZEWOY^28^	2		40.80^d^	0.609^d^	79.75^d^	120.55^d^	13.28^d^
	17	VUKYUX^11^	1	A	4.66	0.195	0.45^c^	5.11	20.70
				B	4.61	0.143	0.02^c^	4.63	
ZnBr_2_	18	VUKYOR^11^	1	A	3.08	0.060	0.63^c^	3.71	3.41
				B	3.41	0.110	0.05^c^	3.46	

a For disordered structures where multiple configurations have been considered, the lowest energy configuration is reported. For the complete set of results see the ESI.†

b The value of Δ*E*_MOF_ is quoted per minimum framework unit, *i.e.* (ZnX_2_)_3_(tpt)_2_, or 81 atoms, rather than per unit cell as quoted in the calculation output, to make the values comparable.

c It is important to note here that although 1*R*-(−)-methyl acetate and vanillin have a non-zero Δ*E*_conf_, geometric comparison with the set of generated conformers shows that the conformer in the experimental structure matches the global minimum energy conformer, and therefore the non-zero energy values are indicative of calculation errors resulting from the convergence tolerances.

d These data values are included to show that calculations on this structure have been carried out, however they will not be discussed as the calculated energies are unusually high and may have resulted from the use of restraints/constraints on the disordered molecule.

The experimental CS structure 10 (CENBUV) contains one dapsone molecule, two disordered n-hexane solvent molecules and two water molecules in the asymmetric unit. Both *n*-hexane solvent molecules are 2-part disordered with equal occupancies, so four configurations of the structure have been considered to account for all combinations of solvent location. The Δ*E*_total_ of the dapsone guest in each configuration are within a 2 kJ mol^−1^ window of each other (see Table S2†). Breaking down the Δ*E*_total_ term into its components, we can see this is solely resulting from variation in intramolecular strain energy Δ*E*_strain_, as all configurations share the same conformation, with a calculated Δ*E*_conf_ = 1.8 kJ mol^−1^. Although it is not large, the difference in energies between configurations shows the impact of the proximity of solvent, and therefore sterics, on the total energy of guest molecules.

In the following sections, the impact of the CS environment on the energy and conformation of the guest molecules will be discussed. Any analysis of interactions between the guest and the framework or other guests will be commented on from a qualitative perspective; detailed quantitative discussion, similar to recent work by Carroll,^27^ would be required to fully understand the strength of the interactions.

The structure of doxorubicinone (16, EZEWOY) has unphysically high Δ*E*_strain_ and Δ*E*_conf_ energies. The host framework is well-resolved, however the guest model required a considerable number of restraints and constraints to impose reliable geometry and crystallographic parameters. There is a high likelihood that these have significantly distorted the molecular geometry and resulted in these artificially high calculated energies. For this reason this structure is an outlier in the group and has not been included further in this analysis. All remaining discussion includes only the remaining 16 CS structures.

### 3.1 Total relative energy

The distortion of any molecule away from its gas phase geometry involves an increase in the intramolecular energy Δ*E*_strain_ and possibly of the conformational energy Δ*E*_conf_, if the molecule adopts a conformer other than its most favourable gas phase conformation within the CS. Firstly the total relative energy Δ*E*_total_ of a molecule in a CS crystal structure from its gas phase geometry is analysed. This is calculated as the difference between the energy of the isolated molecule in the geometry from its DFT optimised crystal structure and the gas phase (global minimum) energy, according to eqn (3).

**Fig. 3:**
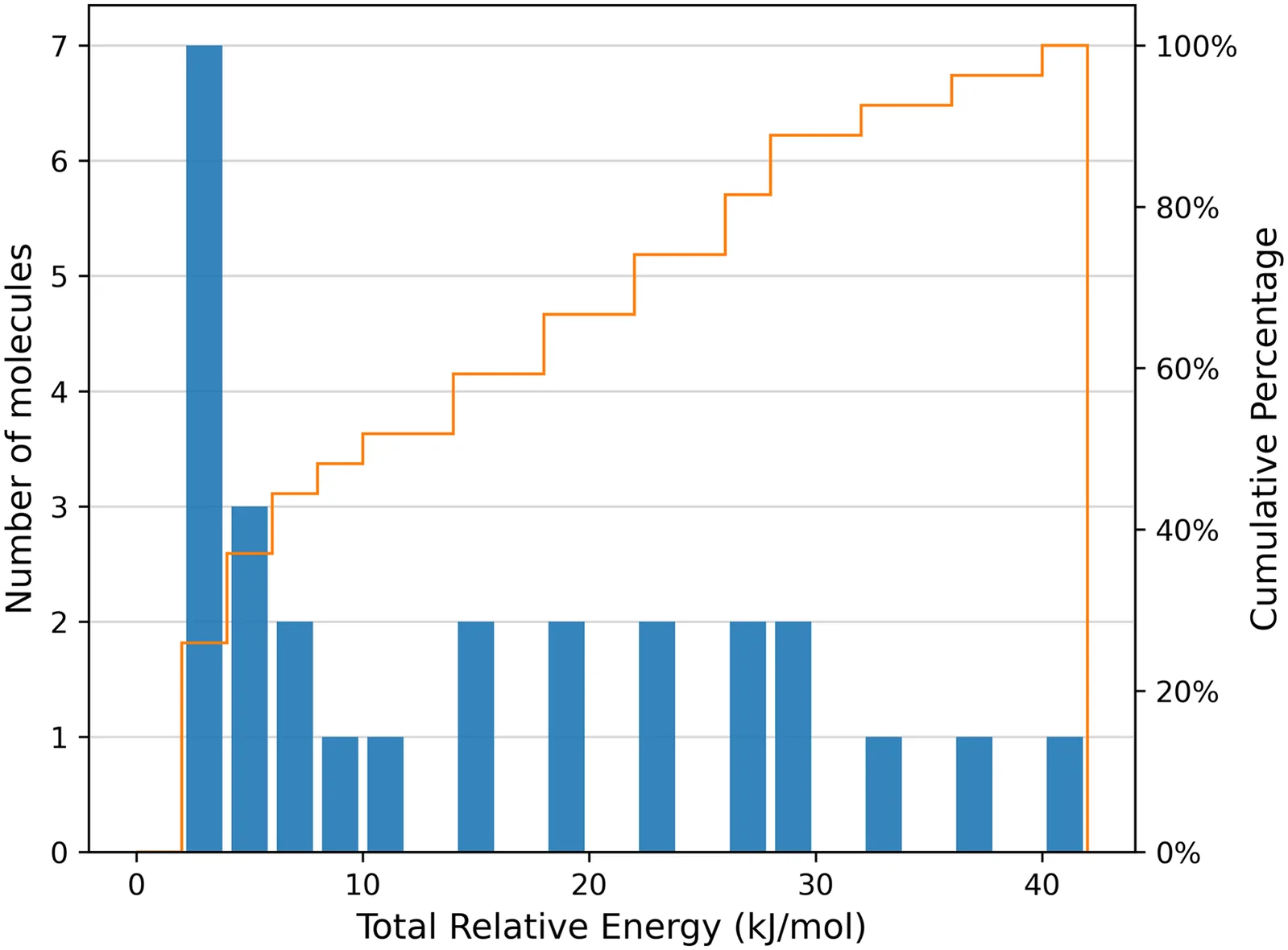
Histogram detailing the distribution of the total relative energy Δ*E*_total_ of the 27 distinct molecular geometries of the 15 unique guests in the 16 CS structures chosen, with a cumulative percentage of the distribution of molecules as a function of strain energy (orange line). Histogram bin width = 2 kJ mol^−1^.

The distribution of Δ*E*_total_ is detailed in Fig. 3 for the 27 distinct guest geometries exhibited in the 16 optimised experimental CS structures (excluding structure 16) outlined in Fig. 2. The distribution includes the total relative energies of each independent molecule found within each crystalline sponge structure.

**Fig. 4:**
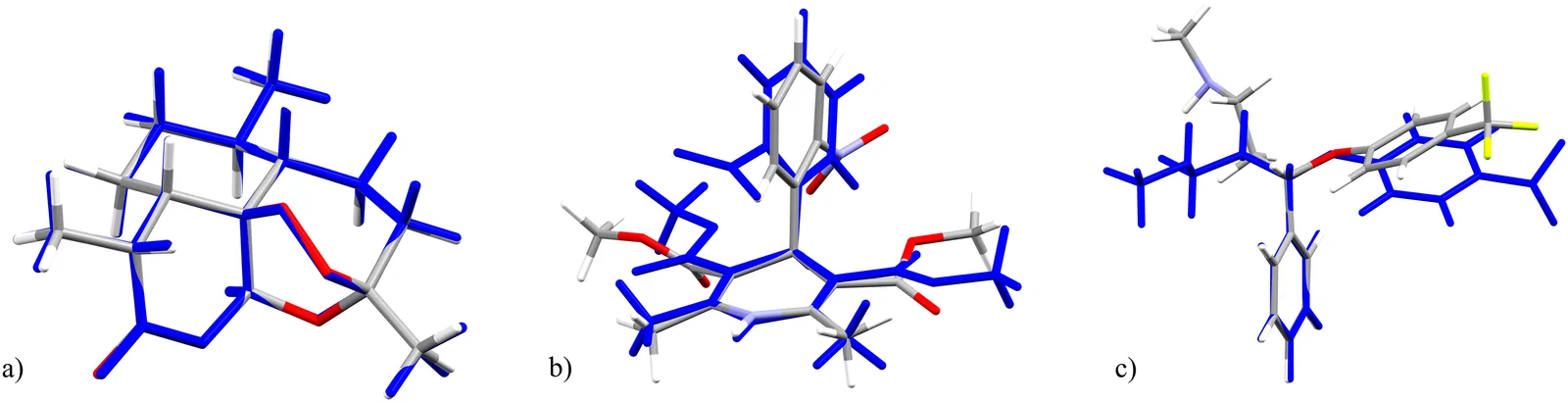
Overlays of the crystalline geometry of (a) (+)-artemisinin (2), (b) nifedipine (11) and (c) fluoxetine (6) in the optimised CS structure (element colours) with its gas phase (global energy minimum) conformer (blue).

Molecules which would typically be considered rigid are slightly distorted and therefore have a non-zero total relative energy: we calculate values of Δ*E*_total_ = 2.50 kJ mol^−1^ for 4-trifluoromethylphenyl azide (5, XAZPAS), and 3.99 kJ mol^−1^ for (+)-artemisinin (2, XAZPEW), which has zero rotatable bonds, excluding terminal methyl groups (Fig. 4a). Although the geometric difference is small, this demonstrates the crystal packing effects present in CS structures induce distortions of even the most rigid of molecules. The lowest calculated Δ*E*_total_ is 2.41 kJ mol^−1^ for one of the four vanillin guest molecules in 3 (XAZNUK), and one third (9 of 27) of molecules studied have a calculated Δ*E*_total_ less than 5 kJ mol^−1^ (approximately 2RT at room temperature). These mostly correspond to the smaller molecules with few rotatable bonds (Table 1): structures 2, 3, 4 (ZOQSUV), 5, 17 (VUKYUX), and 18 (VUKYOR).

**Fig. 5:**
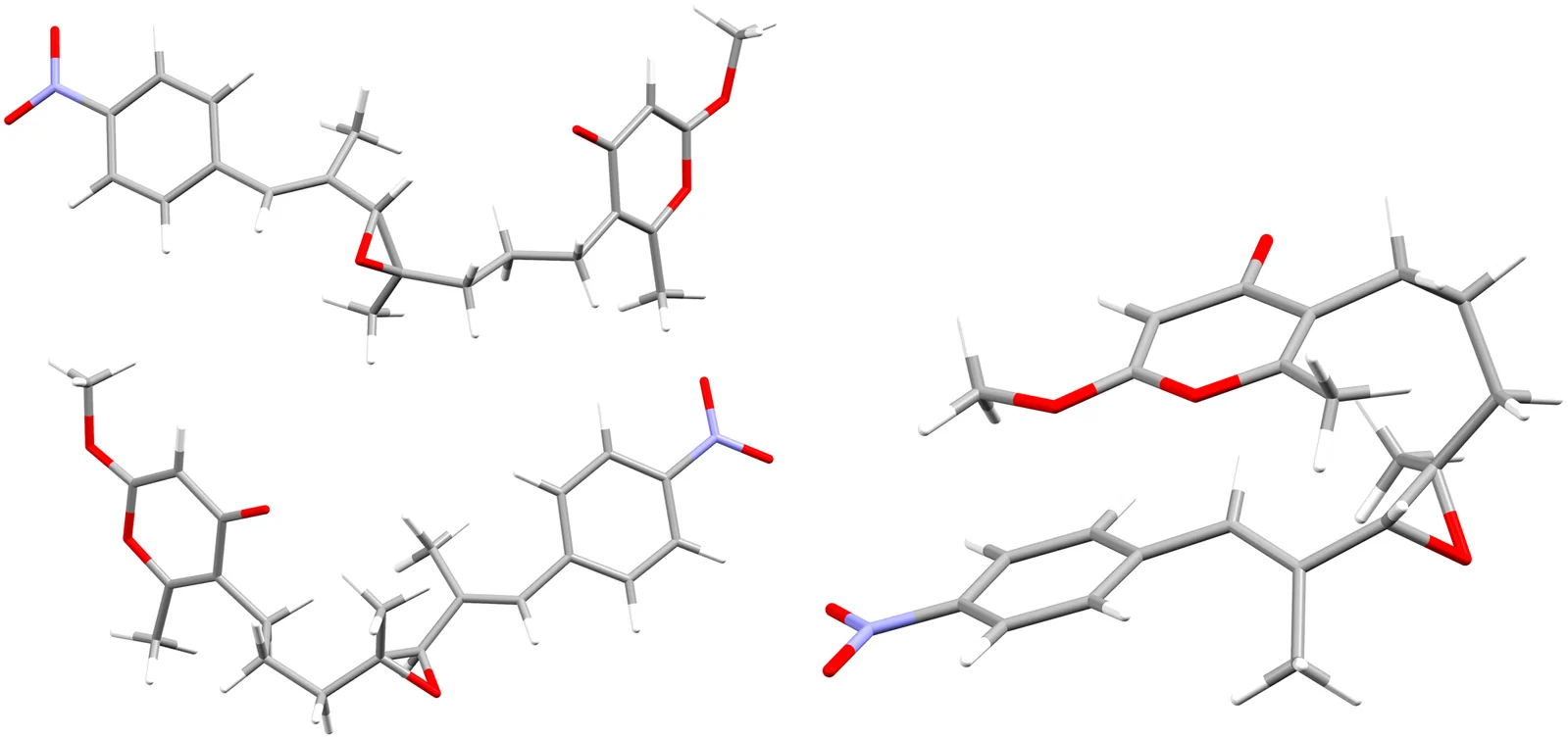
The two unique crystalline molecular geometries of tenebrathin in the pores of the optimised CS structure 13 (left) and the gas phase (global energy minimum) structure of tenebrathin (right).

The conformations of larger molecules with more flexible degrees of freedom are more substantially influenced by the CS environment. Ignoring the outlier, doxorubicinone (16), calculated Δ*E*_total_ of guests in CS structures range up to 41 kJ mol^−1^. Examples of the highest energy guests include nifedipine (11 RECJAN, Δ*E*_total_ = 32.7 kJ mol^−1^), fluoxetine (6 CENBOP, Δ*E*_total_ = 26.5 kJ mol^−1^), and tenebrathin (13 COQBIW, Δ*E*_total_ = 41.0 and 36.2 kJ mol^−1^ in the two distinct guest sites), which all show significant geometric distortion from their gas phase conformation in the CS (see Fig. 4b, c and 5).

From the perspective of understanding the impact of the CS on the conformation of a guest molecule, these results demonstrate that the interactions with guest molecules in the pore environment often play a role in dictating their molecular conformation: there is always some distortion of molecular structures determined using the CS method away from the intrinsically most stable geometry of the isolated molecule. While this can result in small energetic changes in the structures of small molecules, the effect is often large and should be considered when interpreting CS structures.

We analyse Δ*E*_total_ in more depth in the following sections, where the distortion is broken down into conformational change Δ*E*_conf_ and intramolecular strain Δ*E*_strain_.

### 3.2 Conformational energy: which conformation is adopted in CS structures?

**Fig. 6:**
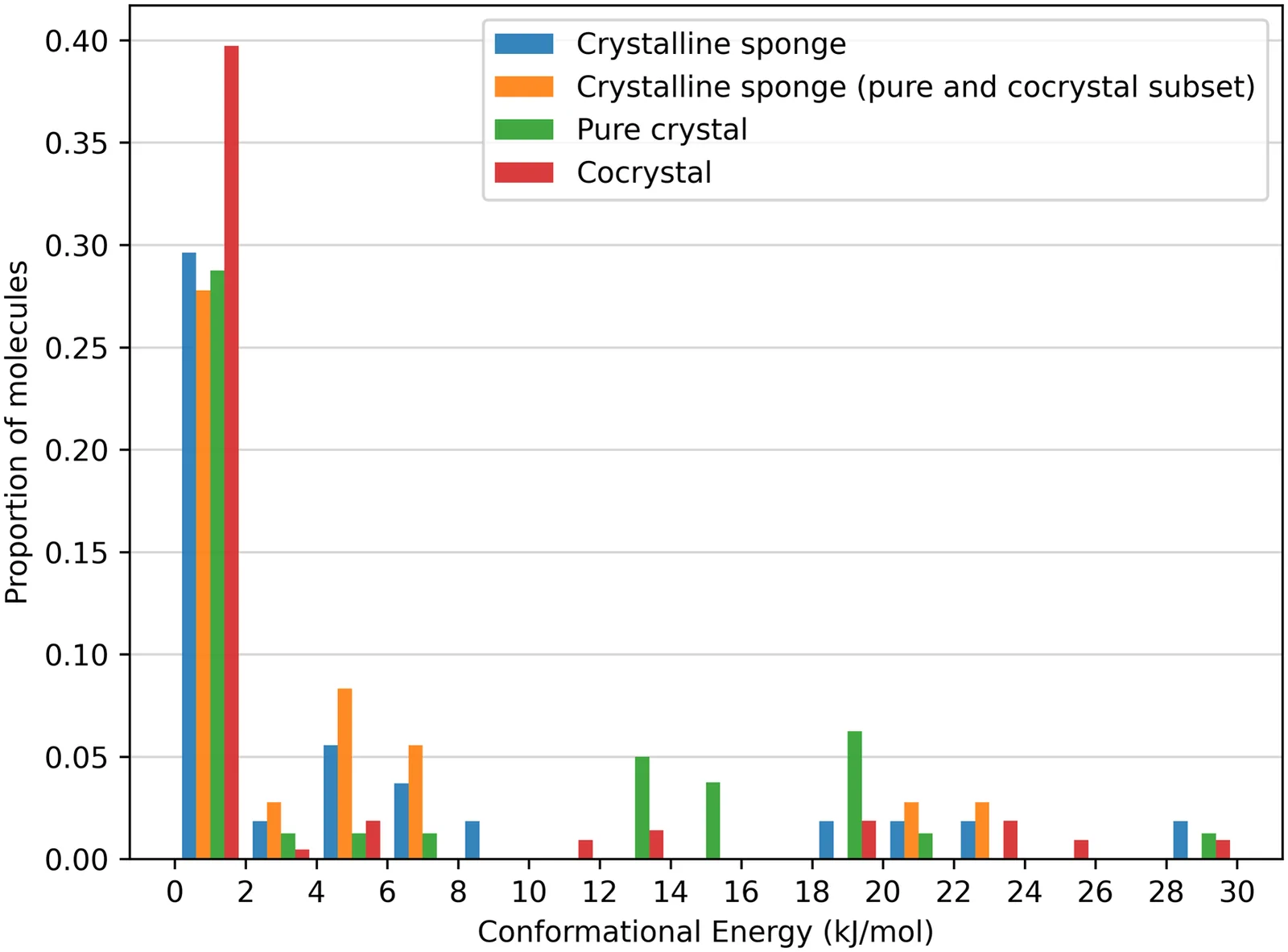
Histogram detailing the distribution of conformational energies of the guest molecular geometries in all CS structures (blue), subset of CS guests with pure and cocrystal structures (orange), pure crystal (green) and cocrystal (red) structures studied. Histogram bin width = 2 kJ mol^−1^.

During crystallisation, molecules are not restricted to their lowest energy conformer, and the balance of inter- and intramolecular interactions can sometimes lead to higher energy conformers.^30^ We quantify this with Δ*E*_conf_, the difference in energy between the conformer that relates to the crystalline molecular geometry and the global minimum energy conformer found by the conformational search process (see Fig. 1). The distribution of conformational energies of guest molecules in CS structures, and comparison to those in pure and cocrystal structures, is detailed in Fig. 6.

For all molecules, a corresponding conformer to that obtained from the experimental crystal structure is found in the generated DFT conformer landscape. Most guest molecules do not adopt the global minimum energy conformer in their CS structures: only 6 out of 16 CS structures contain a guest molecule which adopts the global energy minimum conformer. (+)-artemisinin (2), 4-trifluoromethylphenyl azide (5), and the dithiine guest (7, GARLAQ), all of which have Δ*E*_conf_ = 0 kJ mol^−1^, are reasonably rigid molecules. Three further structures containing vanillin (3) and (1*R*)-(−)-menthyl acetate (17 and 18) adopt the global minimum conformer in their experimental CS crystal structures; however, these have small, non-zero (up to 0.6 kJ mol^−1^) calculated Δ*E*_conf_, which is considered negligible once a visual comparison is made with the corresponding global minimum energy conformer. These small energetic discrepancies provide a measure of the numerical noise in the molecular optimisations applied in this work.

Although it is rare for molecules to adopt their lowest energy conformer in CS structures, for over half of the unique molecules (59%, 16 out of 27 molecules in all structures) the crystalline geometry remains close to the global minimum (below 2.0 kJ mol^−1^). In 23 out of 27 unique guest molecules, the conformational energy lies below 10 kJ mol^−1^: molecules usually adopt low energy conformers in CS structures.

**Fig. 7:**
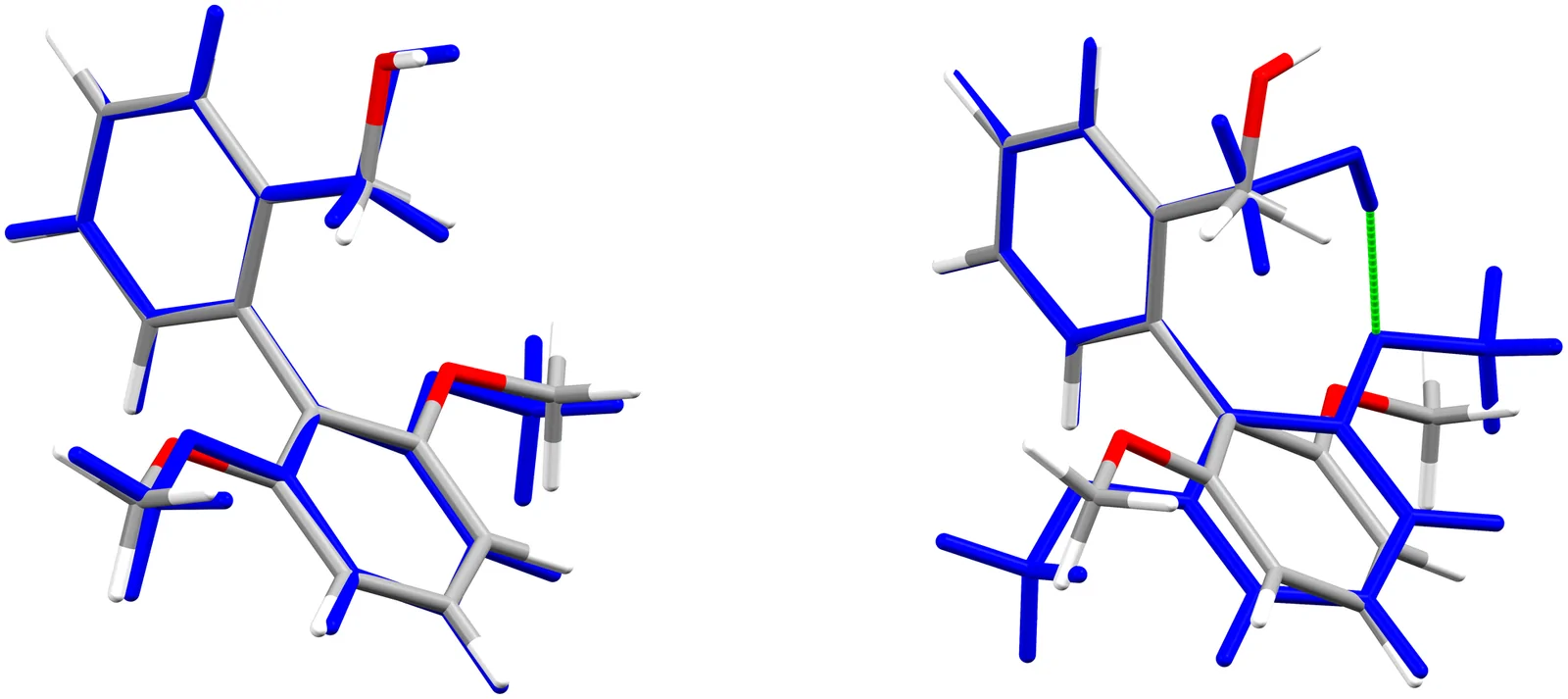
Overlays of the crystalline molecular geometries (element colours) of the guest molecules in CS structure 9 with their respective local conformers (blue). Intramolecular hydrogen bonding is indicated with a green line (right).

For the remaining molecules, their geometry in the CS structures corresponds to a conformer that is energetically far above the gas phase global energy minimum. Structures 8 (LIRLIL) and 9 (LIRLEH) each contain a biaryl guest with high Δ*E*_conf_, at 21.78 and 21.05 kJ mol^−1^, respectively; both CS structures also contain a second guest with much lower Δ*E*_conf_ (see Table 1). In the gas phase, the global minimum conformer for the guest molecules in both 8 and 9 adopt a geometry stabilised by intramolecular hydrogen bonding. The biaryl guests which adopt a local conformation with no intramolecular hydrogen bonds have a high Δ*E*_conf_, due to the high energetic barrier for geometric distortion/destabilisation of the molecule. The biaryl guests with low Δ*E*_conf_ adopt geometries with intramolecular hydrogen bonding intact after relaxation to their local conformation (see Fig. 7). Both guest molecules of tenebrathin (13), as mentioned previously in discussion of Δ*E*_total_, also adopt relatively high energy conformers in their CS structure, with Δ*E*_conf_ = 19.98 and 28.45 kJ mol^−1^.

**Fig. 8:**
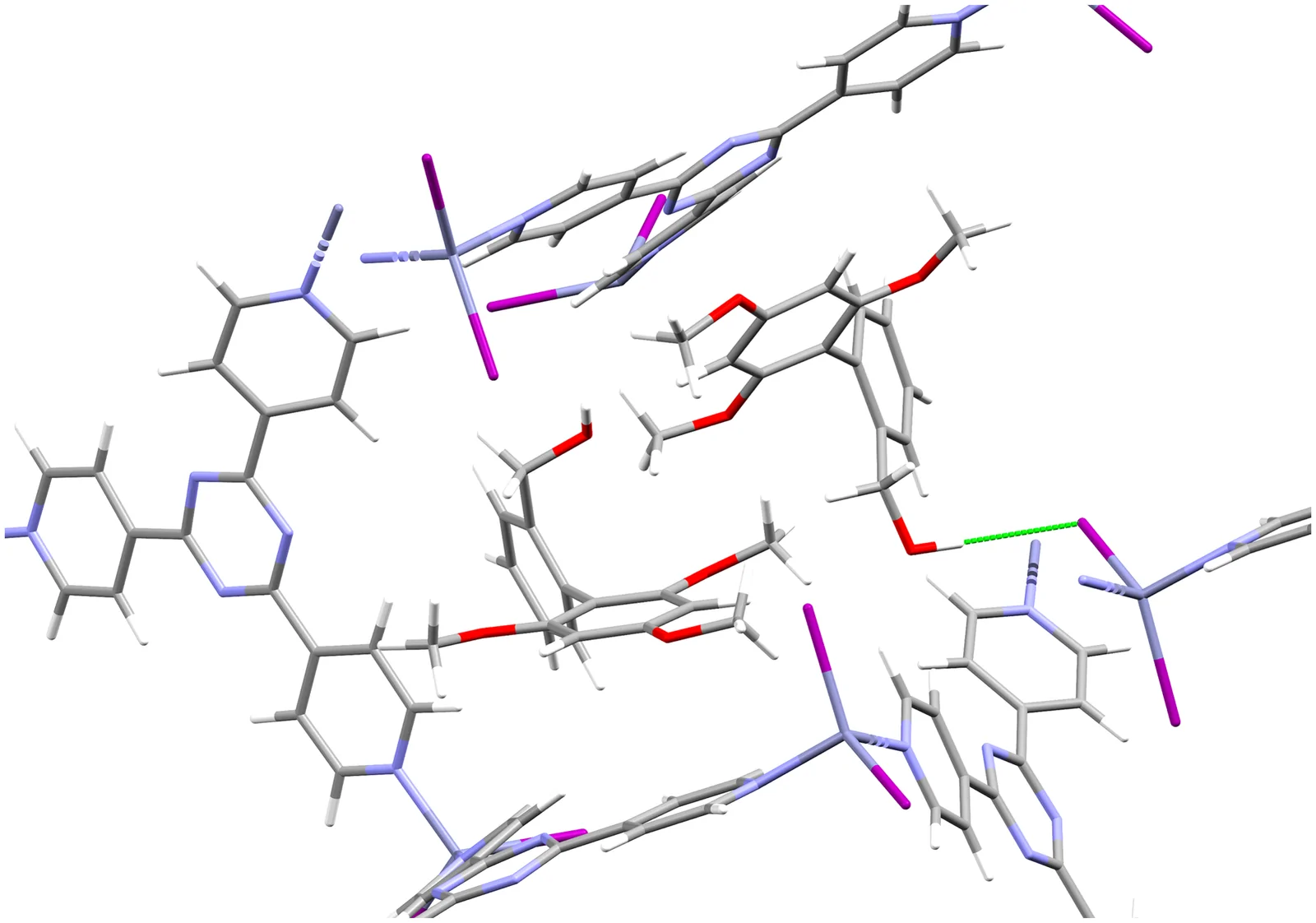
The two unique guest molecules in the pores of CS structure 8. The highlighted guest–framework interaction relates to the guest with high strain energy, a result of distortion of the local conformer away from intramolecular hydrogen bonding.

Previous work by Thompson^30^ showed that conformers with large surface areas are favoured in the crystal structures of flexible molecules, as it provides greater available surface area for stabilising intermolecular interactions, while lower energy conformers typically display relatively compact geometries to enable intramolecular stabilisation. This is what we observe for tenebrathin, which adopts extended conformers in its CS structure, compared to the folded global energy minimum conformer (Fig. 5), that could be stabilised by general van der Waals interactions with solvent and the framework. This is not the case for high Δ*E*_conf_ values in 8 and 9, however, where the driving force for these molecules comes from breaking intramolecular hydrogen bonding stabilised by strong, specific interactions with the framework (Fig. 8).

### 3.3 Intramolecular strain energy

The distortion of any molecule in a crystal structure away from the relaxed isolated molecular geometry involves an increase in the intramolecular energy, *E*_strain_. This energy is calculated as the difference in energy of the molecule in the optimised crystal structure from its local energy minimum on the conformational energy surface (Fig. 1).

**Fig. 9:**
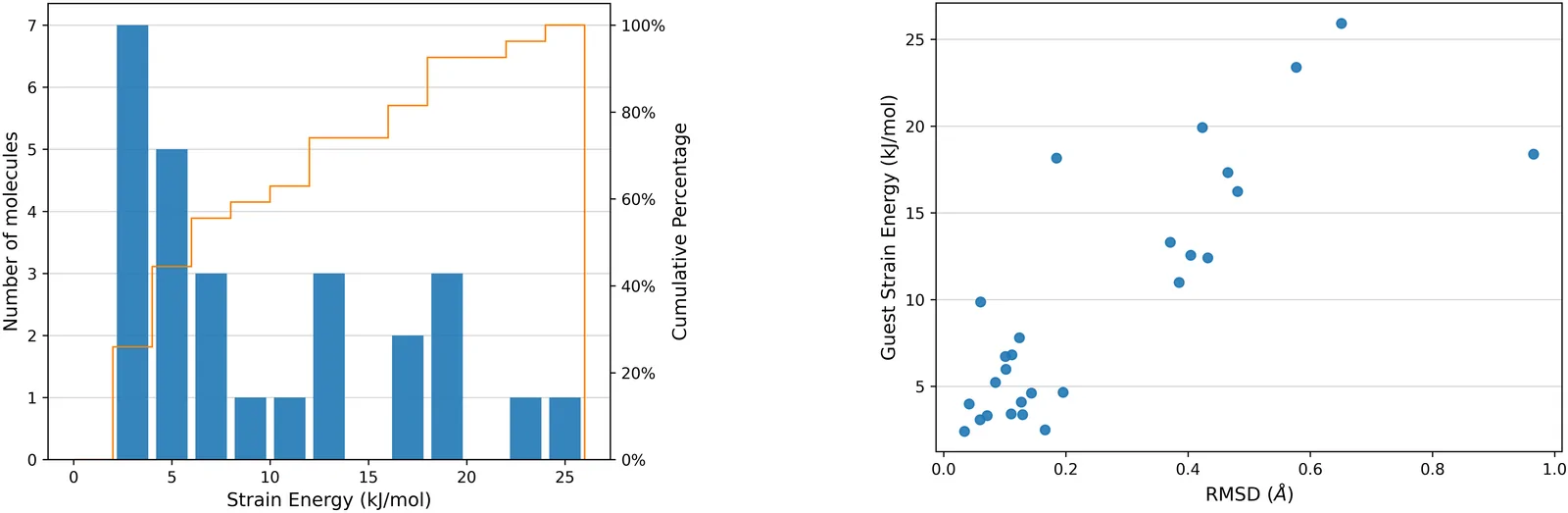
Left: Histogram detailing the distribution of intramolecular strain energies of the CS guest molecules studied, with a cumulative percentage of the distribution of molecules as a function of strain energy (orange line). Right: Plot of Δ*E*_strain_ against RMSD of atomic positions between the optimised crystalline molecular geometry and the conformation of the optimised isolated molecule of the CS guest molecules studied. Histogram bin width = 2 kJ mol^−1^.

The distribution of Δ*E*_strain_ is detailed in Fig. 9 for the 27 distinct guest geometries in the 16 optimised experimental CS structures outlined in Fig. 2. The distribution includes the strain energies of each independent molecule found within each CS structure configuration. Calculated CS strain energies range up to 26 kJ mol^−1^ and are concentrated in the lower end of the distribution: over a third (10 out of 26 guest geometries) have calculated Δ*E*_strain_ < 5 kJ mol^−1^ and 59% of guest molecules (16 out of 26 guest geometries) have Δ*E*_strain_ < 10 kJ mol^−1^. These molecules adopt geometries energetically near to a gas phase optimised conformer in the CS structures.

There is a correlation between the intramolecular strain energy Δ*E*_strain_ of the guest within the CS pores and the RMSD in atomic positions between the crystalline guest molecular geometry and the optimised isolated guest (see Fig. 9), confirming that large distortions in the molecular geometry are usually associated with a relatively large energetic cost. There are a few cases where significant geometry changes have a relatively low energetic cost, such as risperidone (14, EZEXAL) which has an extended structure; or where a small change in molecular distortion leads to a high strain energy, such as the dithiine guest (7). The latter example contains two highly-strained 4-membered rings in the molecular structure, for which there is a large energetic penalty for making relatively small geometric changes.

There are several instances of surprisingly high strain energies above 20 kJ mol^−1^. The highest values calculated from the guest molecules studied here are 23.39 kJ mol^−1^ in 8 and 25.9 kJ mol^−1^ in 11. High strain energies are not restricted to the most flexible molecules; two molecules with relatively few flexible degrees of freedom both show a relatively large strain energy in their CS structures: 7 (Δ*E*_strain_ = 18.16 kJ mol^−1^, 2 rotatable bonds) and 10 structure 1 (see ESI,† Δ*E*_strain_ =15.50 kJ mol^−1^, 4 rotatable bonds).

### 3.4 Pure and cocrystal structures

To understand how the CS pore environment differs from typical crystal packing, the conformational energetics of molecules in their pure and cocrystal structures have been compared to those calculated for the CS structures. The CS method is used primarily for structure elucidation of substances that do not crystallise or are produced in minute quantities, so several guest molecules in this study do not have any non-CS crystal structures which can be studied. Of those that do have crystal structures, there are 10 molecules with pure (single-component) structures and 7 molecules with cocrystal structures, including 6 which have both pure and cocrystal structures. The complete set included in this study is detailed in Tables S3 and S4.†

**Fig. 10:**
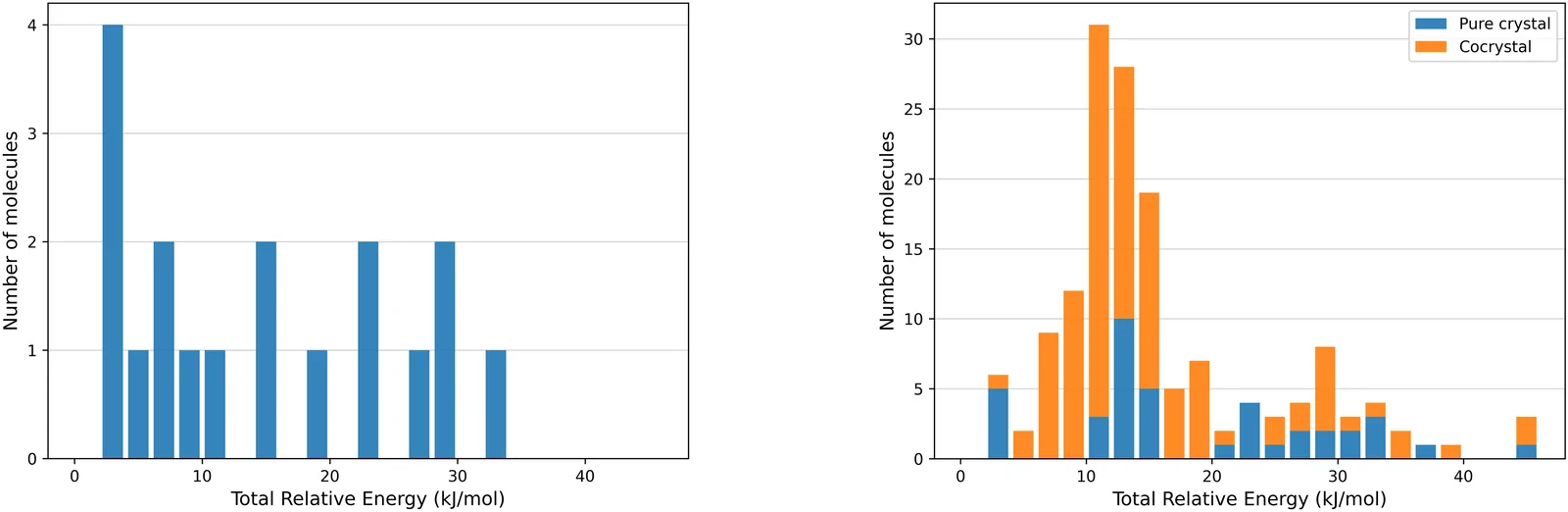
Left: Histogram detailing the distribution of total relative energies Δ*E*_total_ of CS guests with pure and cocrystal structures. Right: Histogram detailing the distribution of the total relative energies Δ*E*_total_ of the pure and cocrystal structures studied. Histogram bin width = 2 kJ mol^−1^.

The distributions of Δ*E*_total_ in conformers present in CS structures are compared to those in pure and cocrystal structures in Fig. 10. Molecules typically have a lower total relative energy in the CS compared to both their pure and cocrystal structure counterparts. The Δ*E*_total_ distribution for molecules in their pure and cocrystal structures ranges up to 45 kJ mol^−1^, higher than those observed in the CS at 34 kJ mol^−1^. For pure and cocrystal structures the distributions peak between 12–14 and 10–12 kJ mol^−1^, respectively, whereas for CS structures this is observed between 2–4 kJ mol^−1^. For the subset of CS structures where the guest has pure and/or cocrystal structures, 47% of unique molecules have a calculated Δ*E*_total_ below 10 kJ mol^−1^. In comparison, the proportion of unique molecules with Δ*E*_total_ below 10 kJ mol^−1^ is much lower in pure and cocrystal structures: 12.5% (5 out of 40) of molecules in pure crystal structures and 20% (23 out of 114) of molecules in cocrystal structures.

The shift to higher Δ*E*_total_ for cocrystals could likely be a consequence of the strong interactions between coformers, which will have an impact on molecular geometry and Δ*E*_total_. The distribution also has a larger Δ*E*_total_ range, suggesting that the intermolecular interactions with the pore environment of the CS are weaker than in typical crystal structures. The Δ*E*_total_ distribution of pure and cocrystal structures in Fig. 10 is also skewed by unequal proportions of cocrystal structures: some molecules have many more structures than others and if the number of structures per molecule of interest were equal, the distribution would be flatter. Fig. S2† shows the Δ*E*_total_ distribution for cocrystal structures, broken down by the molecule of interest: there are significantly more cocrystal structures than pure crystal and CS structures, providing greater sampling of cocrystal structures and resulting in an inconsistent comparison.

As previously described, a large proportion of calculated Δ*E*_conf_ is limited to <2 kJ mol^−1^ and consequently Δ*E*_strain_ has the biggest contribution to Δ*E*_total_ of guest molecules. The distribution of Δ*E*_strain_ for both pure and cocrystal structures is detailed in Fig. S3b.† Calculated strain energies range up to 28 kJ mol^−1^ and peak between 10–12 kJ mol^−1^. Previous conformational studies of single-component organic crystal structures has shown that strain energies range up to 20 kJ mol^−1^ and the majority (75%) of strain energies lie below 10 kJ mol^−1^.^30^ Comparison to the Δ*E*_strain_ distribution of the subset of 10 CS guest molecules that have pure and/or cocrystal shows that the distribution peaks in the 2–4 kJ mol^−1^ bin (Fig. S3a†) and only 61% have a strain energy below 10 kJ mol^−1^ (11 out of 18 unique molecules). Similarly to CS structures, there is a positive correlation between Δ*E*_strain_ and RMS deviation of atomic positions between the experimental and gas phase local conformer molecular geometries, for both pure and cocrystal structures (Fig. S4†).

The increased probability of a higher Δ*E*_strain_ in pure and cocrystal structures suggests that the close-packed nature of typical crystal packing provides greater stability of molecular distortion than interactions in the pores of the CS framework. It should be noted that Thompson's study^30^ excluded molecules with potential for intramolecular hydrogen bonding, while the molecules in this study have greater potential for hydrogen bonding. These strong interactions could also lead to a higher Δ*E*_strain_.

### 3.5 Framework strain

Quantifying the strain of the CS framework gives insight into the breathing behaviour of the interpenetrated structure and how it is affected upon uptake and exchange of guest molecules. All guest and solvent molecules were removed from the DFT optimised CS structures before running single-point energy and geometry optimisation calculations to find the intramolecular strain energy of the framework, Δ*E*_MOF_, as described in section 2.3. Calculated Δ*E*_MOF_ values are reported per minimum framework unit ((ZnX_2_)_3_(tpt)_2_, 81 atoms) and can be found in Table 1. Structure information and cell volume changes are found in Table S5.†

A CS structure containing only solvent was included to understand the impact of a non-interacting guest on the framework. The ZnI_2_ structure (1, LABMOT) was chosen due to the inclusion of cyclohexane, present in many structures in this study. The structure has a calculated Δ*E*_MOF_ of 16.06 kJ mol^−1^ with only a slight increase in cell volume of 0.62% after all solvent molecules are removed, demonstrating that weakly interacting solvent in the pores accounts for some of the induced framework strain, but do not significantly distort the structure.

The CS frameworks have a calculated Δ*E*_MOF_ of up to 21 kJ mol^−1^ per minimum unit, and only 7 out of 16 structures studied have a framework strain higher than the solvent-only structure. Δ*E*_MOF_ is on the same order of magnitude as for single molecules, indicating that breathing of the framework does not come at a large energetic penalty. Furthermore, guest exchange does not cause significant amounts of distortion in the framework: the change in cell volume between the optimised experimental structure and the optimised empty framework is typically less than 3%. This exhibits the breathing capabilities of the framework that occurs during the thermodynamic guest exchange process, and does not appear to correlate with framework strain (Fig. S6†).

The system including dapsone in the ZnI_2_ framework (10) is an outlier in the subset. The guest molecule in the experimental structure is clearly defined, with no constraints or restraints applied, and there is no mention of unusual observations relating to the framework by the authors.^28^ The framework, however, has a calculated Δ*E*_MOF_ = 100.95 kJ mol^−1^, an order of magnitude greater than typical values. In addition, the cell volume contracts by 15.51%, much greater than all other structures. A typical CS structure has a unit cell volume of 15 000–16 000 Å^3^ whereas 10 has a volume of 12 000 Å^3^. The high framework strain could result from strong interactions between the guest molecule and the framework, but would require separate detailed studies to understand fully. Comparisons of the structure before and after re-optimisation without guests and solvent can be found in Fig. S6–S8 in the ESI.†

Insight into the effect of changing the halide ion within the framework can be gained from comparison of ZnCl_2_ and ZnBr_2_ analogues containing the same guest molecule. The experimental structures 17 (ZnCl_2_) and 18 (ZnBr_2_) containing 1*R*-(−)-menthyl acetate are included in this study. The 1*R*-(−)-menthyl acetate guest molecules are present in the same exchange sites in both structures, with additional presence of one and two CHCl_3_ solvent molecules, respectively. Calculated framework strain values show a large difference between each system: Δ*E*^av^_MOF_ = 3.41 kJ mol^−1^ for ZnBr_2_, with an overall cell volume increase of 0.05%, and Δ*E*^av^_MOF_ = 20.7 kJ mol^−1^ for ZnCl_2_, with an overall cell volume reduction of 6.27%. The ZnCl_2_ structure also has an overall smaller experimental unit cell size than the ZnBr_2_ structure (15 720.4 Å^3^*versus* 16 176.6 Å^3^). It is clear that guest exchange causes greater distortion of the ZnCl_2_ framework due to smaller overall pore volumes, resulting in higher framework strain.

The average framework strain for the ZnCl_2_ systems (Δ*E*^av^_MOF_ = 16.74 kJ mol^−1^) is larger than for the ZnI_2_ systems (Δ*E*^av^_MOF_ = 11.41 kJ mol^−1^).‡‡We have excluded structures 10 and 16 from averaging values due to their unusually high respective framework and guest energies. There are several factors which could have caused this: the size of the guest molecules in the pores, and the overall unit cell and therefore pore size. In this study, the average molecular weight of guest molecules in ZnCl_2_ structures (296.8 g mol^−1^) is larger than in ZnI_2_ structures (245.0 g mol^−1^). The average unit cell sizes of ZnCl_2_ systems is also larger than ZnI_2_ systems, at 14 335.1 Å^3^ and 15 793.9 Å^3^, respectively. While the increased strain in the ZnCl_2_ frameworks could simply be due to the larger molecules present in the pores, accounting for the average unit cell sizes of the ZnCl_2_ and ZnI_2_ structures indicates that the higher Δ*E*^av^_MOF_ of ZnCl_2_ systems is affected by a combination of factors. The reduced pore size of the ZnCl_2_ system results in greater distortion of the framework to accommodate the overall larger guest molecule size, ultimately leading to higher framework strain.

### 3.6 What is the effect of multiple guests in the pore?

It is not uncommon for guest molecules to soak into several exchange sites within the pores of the CS. There are 8 CS structures in this study which feature multiple guests in the asymmetric unit of the unit cell. Energetic differences between guest molecules in the same CS structure are observed, resulting from molecules experiencing different interactions in each exchange site. For all but one CS structure with multiple guests, ΔΔ*E*_total_ is below 5 kJ mol^−1^ (Table 1), but is much larger, ΔΔ*E*_total_ = 11.68 kJ mol^−1^, in the CS structure of vanillin (3).

The intermolecular interactions experienced by the molecule, resulting from differences in pore environment, can have a profound effect on Δ*E*_strain_ and Δ*E*_conf_. To understand how molecular geometry is affected by multiple guests present in the pore, we can look at structures 8 and 9. Each structure has two guest molecules: guest A with high Δ*E*_strain_ and low Δ*E*_conf_, and guest B with low Δ*E*_strain_ and high Δ*E*_conf_ (see Table 1). The high Δ*E*_strain_ of guest A and high Δ*E*_conf_ of guest B results from distortion that breaks the intramolecular hydrogen bond within the molecule; in the case of guest A, it is simply intramolecular strain/distortion, whereas for guest B, the complete change in geometry to a conformation with no intramolecular hydrogen bonding is energetically costly (see Fig. 7). The guest with high Δ*E*_strain_ in each structure relaxes to a local conformational minimum stabilised by intramolecular hydrogen bonding. The number and type of interactions present in these two structures were recently discussed in a systematic study.^27^ In structure 8, interactions between guest A and the framework seem to be dominated by polar O–Ar interactions, while guest B experiences mostly aromatic Ar–Ar, in addition to the long-range dispersion interactions present in the pore environment. Guest–guest interactions between A and B are also observed, which will contribute to the overall molecular strain and conformational changes from the lowest energy conformer.

The experimental CS structure of risperidone in the ZnI_2_ framework (14) contains two guest molecules in the asymmetric unit and enables a quantification of the effect of multiple guests in the pore. Guest B has an increased induced intramolecular strain compared to guest A (Δ*E*_strain_ = 18.39 kJ mol^−1^ and 12.46 kJ mol^−1^, respectively), which is most likely due to experiencing stronger interactions in its exchange site. Configurations where only one molecule of risperidone is present have also been considered to investigate the interactions between guests. The calculated Δ*E*_conf_ of guest A and B is the same in all configurations, regardless of the total number of guests present. When considering only guest B in the pore, the strain energy is unaffected: the calculated Δ*E*_strain_ of 18.13 kJ mol^−1^ in configuration 3 (see Table S2†) is just 0.26 kJ mol^−1^ less than configuration 6 (Table 1), where both guests A and B are present, corresponding to a small RMS deviation in atomic positions of 0.505 Å. When considering only guest A in the pore, however, the conformer in configuration 2 has a calculated Δ*E*_strain_ of 8.26 kJ mol^−1^. Compared to configurations where both guests are present the RMS deviation of atomic positions is small (0.166 Å), but an increase in Δ*E*_strain_ of 4.20 kJ mol^−1^ is observed. While there are no guest–guest close contacts in the experimental and optimised structures, the increase in intramolecular strain energy of guest A when guest B is present demonstrates the influence of long-range interactions between molecules in the pores, which could be direct long-ranged interactions (*e.g.* electrostatics) between guests, or indirect interactions *via* the influence of guest molecules on solvent molecules.

It is clear from these examples that the number of interactions a guest experiences can have a pronounced effect on the intramolecular strain energy of the guest molecules when there are multiple guests in the pore.

## 4 Conclusions

This computational study provides important insights into the influence of the crystalline sponge (CS) environment on guest molecule conformation, which should inform the interpretation and use of molecular geometries that are obtained from CS structures.

Guest molecules in CS structures, particularly larger molecules with several rotatable bonds, almost always experience some distortion away from their most stable gas phase geometries; total relative energies, Δ*E*_total_, calculated as the difference between the geometry in the CS structure and the global energy minimum of the isolated molecule, are frequently small (below 5 kJ mol^−1^ in a third of structures studied here), but range up to 41 kJ mol^−1^. Partitioning these conformational energetics into the conformer energy of the nearest gas phase conformer to the geometry in the CS structure, and strain energy of molecule (distortion away from the nearest local minimum), provides additional insight into the conformational effects of the CS environment on guest molecules.

The largest energetic effects (highest Δ*E*_total_) usually correspond to breaking of strong non-bonded intramolecular interactions, such as intramolecular hydrogen bonds or stabilising interactions from folding of long, flexible molecules, that are present in the most stable geometry of the isolated molecule. In the absence of such effects, molecules in CS structures usually adopt a geometry that is energetically close to one of their lowest energy conformers, although this is often not the lowest possible conformer of the isolated molecule.

By comparing the conformations of guest molecules in CS structures with those in pure, close-packed crystal structures and co-crystals of the same molecules, we find that the molecular geometries from CS structures have a lower total relative energy, and lower molecular strain, than those found in conventional crystal structures. These results suggest that CS structures represent a more “neutral” environment for studying the geometry of organic molecules than conventional crystal structures, providing a closer representation of molecules' intrinsically preferred geometries.

## Author contributions

E. M. Soper: methodology, investigation, analysis, writing. S. J. Coles: supervision, analysis, writing. G. M. Day: conceptualisation, supervision, methodology, analysis, writing.

## Conflicts of interest

There are no conflicts to declare.

## Supplementary Material

CE-027-D4CE00699B-s001

## Data Availability

The data supporting this article have been included as part of the ESI.† Further data, including optimised versions of crystal structures and conformers are available at https://www.pure.soton.ac.uk at [https://doi.org/10.5258/SOTON/D3370].
