# Supplementary material for: The influence of the crystal sponge framework on guest molecule conformation
Source: CrystEngComm. 2025 Jan 31;27(10):1491–502. doi: 10.1039/d4ce00699b (PMC13583525; doi:10.1039/d4ce00699b)
Supplement: Supplementary file 1 [file CE-027-D4CE00699B-s001.pdf]

Supplementary Information (SI) for CrystEngComm.
This journal is © The Royal Society of Chemistry 2025

Supporting Information: The influence of the crystal sponge framework on guest molecule conformation

Eleanor M. Soper, Simon J. Coles, Graeme M. Day 9th

January 2025

1 Methods

1.1 Geometry Optimisations

For consistency, the same level of theory is used for all crystal structure and single molecule en- ergy calculations. All dispersion-corrected density functional theory (DFT-D3) calculations were performed in the CRYSTAL17[1] software as it allows the consistent use of atom-centred basis sets for both crystalline and molecular systems. The POB-TZVP basis set[2, 3] and B3LYP func- tional[4] are used in combination with the D3 dispersion correction[5]. To account for the basis set superposition error (BSSE) that arises in finite basis sets, the geometric counterpoise (gCP) correction[6] is implemented in all calculations. Since the gCP implementation in CRYSTAL17 is parameterised for 4th-row and higher elements, iodine is internally substituted with bromine by the program.[7] For periodic structures, shrinking factors of 3 and 3 were used for the Monkhorst Pack and Gilat nets for consistent sampling of reciprocal space.

All geometry optimisations in this study were performed with internal redundant coordinates to reduce the number of optimisation steps required for convergence. The INTLMIXED keyword used performs optimisations in a mixed coordinate system and is convenient for larger numbers of atoms, as the number of parameters generated is substantially smaller than those generated by the standard internal redundant coordinate optimisation setting (INTREDUN) in CRYSTAL17.

Convergence criteria for all optimisations were kept as the default settings in CRYSTAL17. Thresholds for convergence on RMS gradient, RMS displacement, maximum gradient and maximum displace- ment were set to 3×10^{-4}, 1.2×10^{-3}, 4.5×10^{-4} and 1.8×10^{-3} a.u., respectively. The threshold in energy change between optimisation steps was set to the default, at 1×10^{-7} a.u. No constraints were imposed on the atomic positions or unit cell parameters, so all systems were fully relaxed dur- ing geometry optimisations. All single molecule calculations were also performed in CRYSTAL17 using the same functional, basis set, DFT and optimisation settings as for periodic lattice energy minimisations.

1.2 Conformational Searches

Using a conformer generation and clustering script implemented in our in-house CSPy code, we applied the CREST conformer search[8] method to generate complete sets of conformers for each guest molecule studied. Through an iterative process, CREST samples conformational space using metadynamics simulations to generate ensembles of minimum-energy structures on the potential energy surface. Descriptors for total energy, rotational constants and RMSD are used to distinguish between generated conformers and rotamers, resulting in a conformer-rotamer ensemble (CRE). Our in-house code generates multiple CREST searches from diverse starting structures to fully search the conformational landscape. Duplicates from the combined CREs are removed by cluster- ing based on torsion angles within a maximum energy window of 2 kJ/mol. The CREST energy window for conformers is 37.656 kJ/mol (9 kcal/mol).

All unique conformers resulting from the initial force field search were re-optimised with DFT- D3, applying the same computational parameters as used for the crystal structures and crystalline molecular geometries described in Section 1.1. The result should be a complete set of low en- ergy conformers for each molecule. The DFT-optimised conformers were re-clustered to remove duplicates, resulting in the final DFT conformer landscape.

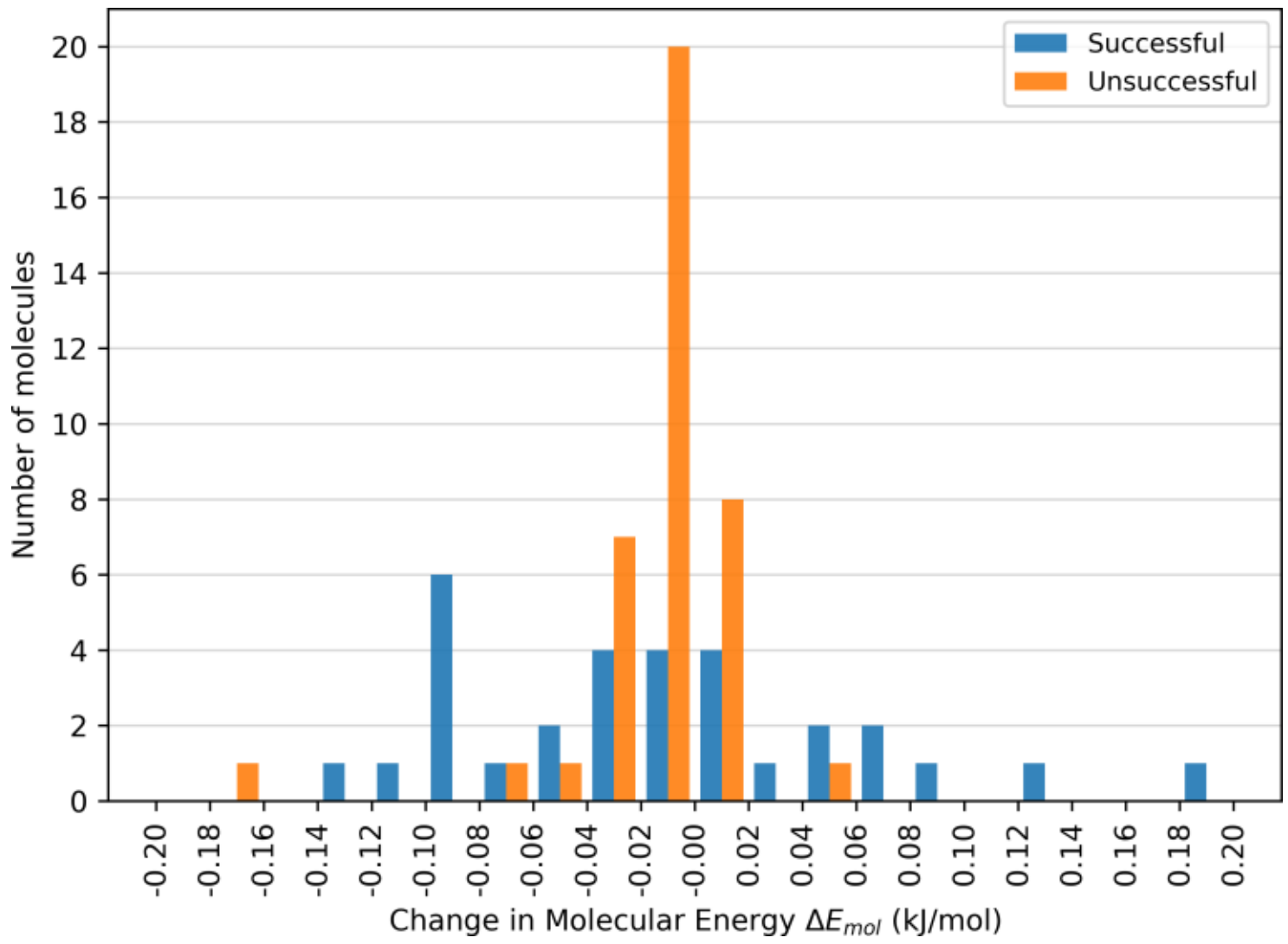

Figure 1: Histogram detailing the distributions of the changes in molecular energy of the conformers that initially had imaginary frequencies at their optimised geometry. The blue bars are those where the imaginary frequency could be removed. The orange bars are the conformers where no attempts could find a re-optimised geometry with no imaginary frequencies.

1.3 Stability of Conformers

To verify that the optimised molecular geometries were at a minimum on the potential energy surface, frequency calculations were carried out at the same level of theory as the geometry op- timisations. For each molecule, frequency calculations were carried out using CRYSTAL17 on the global energy minimum on the conformational landscape and on the local minimum energy molecular geometries obtained by geometry optimisation of geometries taken from experimental crystal structures. Due to the quantity of conformers, frequency calculations were not run on the other conformers, as these conformers are unlikely to change any of the energy differences that we have analysed.

Frequencies for the DFT optimised local and global minimum molecular structures were calcu- lated using the same level of theory as for geometry optimisations described in Section 1.1, with the addition of keywords FREQCALC followed by PREOPTGEOM included in input block 1 (geometry). The molecules must be re-optimised before the frequency calculation occurs as the default convergence tolerances in geometry optimisation before frequency calculations are different than those adopted in normal optimisations. This is to obtain more accurate geometries at the minima and provides more reliable computation of frequencies and modes.[1]

There were 75 molecular conformations with imaginary frequencies identified, out of a total 213 molecular conformations across all crystal structures and global minimum conformers. Various at- tempts were made to re-relax these conformers to stationary points with no imaginary frequencies: each of the 75 conformers was distorted away from the original point using four different methods, followed by re-optimisation: 1) displacements of a range of magnitude, determined by scanning along the selected normal modes where the imaginary frequency is present; 2) random displace- ment applied to all atoms (0.1 Å and 0.2 Å), 3) manual distortion along the normal model using the atomic coordinate displacements reported in the calculation output, and 4) re-optimisation of the molecule using different software (Gaussian09). Scanning of the selected normal modes enables exploration of the potential energy surface to evaluate the minimum and was carried out by includ- ing the keyword SCANMODE plus scan settings (interval and step size) in the FREQCALC input block as outlined in the CRYSTAL17 documentation. After distorting the molecule by any of the above methods, the molecules were re-optimised using the same level of theory in CRYSTAL17 to find the true conformational minimum structure, but with the tighter tolerances used in when preparing frequency calculations.

Of the 75 conformers identified with imaginary frequencies at their original optimised geometry,

42 successfully relaxed to a stable conformation with no imaginary frequencies in at least one of the distortion methods. The largest energetic change of successful distortions was -0.181 kJ/mol, with an average energy change of -0.018 kJ/mol across all categories. A few of the conformers increased in energy by a small amount, suggesting that these changes are in the level of noise in the DFT energy calculations. Changes of this magnitude have an insignificant effect on the energy differences being studied.

Re-optimisations of the remaining 33 conformers were unsuccessful in all attempts. We conclude that these remaining conformers are on flat regions of the energy surface and that the small re- maining imaginary frequencies cannot be easily resolved, but are expected to have a small influence on any of the energy differences being studied. This was confirmed by visualising energy scans of the normal modes corresponding to the imaginary frequencies, which showed very shallow profiles.

The distributions of the change in energy of the molecules with imaginary frequencies are dis- played in Figure S1.

2 Refinement Statistics

For computational study, it is important to have a structure that is complete and well-resolved to ensure that the model will be as close to the actual experimental structure as possible. The quality of an experimental crystal structure is typically measured based on its R-factor (sometimes called "R_1") and the wR_2 refinement statistics. The R-factor is a measure of the agreement between the refined crystallographic model and the experimental X-ray diffraction data, and helps to inform the selection of small-molecule crystal structures for computational study. When it comes to as- sessing the reliability of CS structures, however, the refinement statistics can be quite misleading. A significant proportion of the electron density in CS structures is contained within the framework; contributions from guest molecules are typically far smaller in comparison, particularly if the guest has low site occupancy. Consequently, the R-factor can indicate a "good" value for the model, but it does not necessarily indicate a well-resolved analyte structure or that the contents of the pore are well-defined. Low occupancy and poorly resolved guests can also introduce larger standard un- certainties on bond lengths and geometries. These may be assessed with CCDC Mogul software.[9] Incorporating standard deviations on geometries is work that is currently being undertaken at the University of Southampton, and will give wider insight on interpreting CS structures.[10] Consid- eration of both metrics is important to keep in mind when assessing any CS structure, regardless of whether it will be used for computational study.

The framework is resolved first in structure refinement, as once this has been done, residual guest and solvent electron density is more readily observed. Where there are inconsistencies in the crys- tal such as low-occupancy guests or conformational differences, disorder can arise, as the observed structure is the spatial average of the entire crystal. There is often disorder of the zinc and ter- minal halide atoms in the framework as they have the highest amount of electron density, and this effect is most commonly observed in CS structures containing iodine atoms.[11] The higher electron count of iodine atoms leads to greater scattering contributions from the host framework, which in turn means that guests and solvent are more difficult to resolve.[12] When it comes to studying three-dimensional polymeric structures like MOFs, particularly porous structures that contain solvent-accessible voids, the solvent and other guest molecules that can reside in these pores can be severely disordered.[13]

Electron density that is visible but cannot easily be modelled will negatively impact the R_1 and wR_2 refinement statistics and lower the accuracy of the model. Due to the nature of many CS structures, residual density from highly disordered guests and solvent molecules is common for these systems. Experimentalists may consider using solvent masking techniques such as SQUEEZE[14] in order to account for electron density which cannot reasonably be assigned. In cases where dis- order is severe, a solvent mask can be a reasonable approximation of reality, and the appropriate application should improve both the accuracy of the rest of the model and the refinement statistics. It should be noted, however, that it is not always appropriate to use masking techniques as it has the potential to hide important structural information.

2.1 Preparation of the Dataset

2.1.1 Framework disorder.

Disorder of the framework, most often observed in the zinc and terminal halide atoms, is treated simply by removing the lowest occupancy disordered halide atom pair/triplet and the associ- ated disordered metal centre(s) from each linker in the structure, leaving the highest occupied atoms/fragment. As the target of interest is the guest, not the framework, multiple arrangements accounting for any framework disorder are not considered in this study.

2.1.2 Guest disorder.

Guest and solvent molecules within a certain distance of the framework can experience positional stabilisation due to intermolecular interactions. Beyond this, there are solvent-accessible voids which can accommodate additional solvent and guest molecules. The lack of positional stabil- isation from insufficient intermolecular interactions in these voids can lead to highly disordered molecules.[13]

Guest molecules located over a symmetry element in the unit cell can be equally situated in either orientation, as each symmetry-generated molecule is crystallographically equivalent.[11] CRYS- TAL17 considers the space group symmetry and imports the asymmetric unit of a structure, therefore applying the full crystal structure symmetry in calculations would lead to overlapping guest molecules when the full unit cell is generated, which is physically unrealistic. To account for disorder of guest molecules over symmetry elements, equivalent positions are evaluated as separate cases, and multiple configurations of the cell are considered in a lower symmetry space group: for example, a cell with $C\,2/c$ symmetry where the guest is situated over the two-fold rotation axis can be converted to Cc symmetry, with two configurations of the same structure.

Multiple guest exchange sites within the pores of the CS are not uncommon, particularly with smaller guests. Some CS structures appear to have multiple guests, which on closer inspection is a result of positional disorder. To determine whether there are multiple guest exchange sites in the pore, symmetry and intermolecular distances must be taken into account to ensure realistic separation between molecules. All structures with multiple guests in this study have exchange sites that are generally well-defined, and disorder with other guests or solvent is only observed where guests are situated over a symmetry site.

Where disorder cannot be resolved by reducing the symmetry of the structure, site occupancies must be considered. If guest molecules are too close together, their respective site occupancies can be assessed and those molecules with the lower occupancy are removed. However, for guests where site occupancies are (near) equal and there are more than one realistic structures, multiple structures are generated which take into account site occupancies and may include multiple config- urations. Where the occupancy of a guest molecule is particularly low, the molecule is likely to be disordered with a solvent molecule on the same site. If the occupancy of the guest is greater than that of the solvent molecule, the solvent is removed from the structure, unless the guest position cannot reasonably coexist with other molecules in the pore that exhibit a higher occupancy.

2.1.3 Solvent disorder.

Resolving solvent molecules in the remaining void space in the pore is important for ensuring a fully assigned structure with the whole volume occupied. Inert hydrocarbon solvents, such as cyc- lohexane, are used in the CS method due to their role in facilitating guest exchange, but they may then remain in the pores and will often be disordered due to a lack of strong, directing interactions. Once the guest exchange sites have been located, the solvent molecules are considered in a similar manner to guest molecules i.e. where site occupancies of two solvent molecules are (near) equal, or symmetry related, then multiple configurations are considered. For solvent molecules that cannot coexist and have different occupancies, the lower-occupancy molecule is removed.

Table 1: Molecular criteria considered during crystal sponge structure selection process for this study. Including the number of rotatable bonds, number of aromatic rings, and Lipinski's Rule of Five for identifying pharmaceutical-like compounds.

	CSD Refcode	Guest molecule	Rotatable bonds[a]	Aromatic rings	MW (Daltons)	XLogP3-AA	H-bond donors	H-bond acceptors
					Lipinski's Rule of Five			
					500	5	5	10
**2**	XAZPEW	(+)-artemisinin	0	0	282.33	2.8	0	5
**3**	XAZNUK	vanillin	3	1	152.15	1.2	1	3
**4**	ZOQSUV	*trans*-anethole	2	1	148.2	3.3	0	1
**5**	XAZPAS	4-trifluoromethylphenyl azide	2	1	187.12	3.7	0	5
**6**	CENBOP	fluoxetine	7	2	309.33	4.0	1	5
**7**	GARLAQ	bis(N-cyclobutylimino)-1,4-dithiin-1,2:4,5-tetracarbo-ximide	2	0	362.42	1.9	0	6
**8**	LIRLIL[b]	BBA-8,10,12-OMe	6	2	274.32	-	1	4
**9**	LIRLEH	BBA-8,12-OMe	5	2	244.29	2.6	1	3
**10**	CENBUV	dapsone	4	2	248.3	1.0	2	4
**11**	RECJAN	nifedipine	6	2	346.3	2.2	1	7
**12**	EZEWUE	nifuroxazide	6	2	275.22	2.1	2	6
**13**	COQBIW	tenebrathin	8	2	399.4	4.2	0	6
**14**	EZEXAL	risperidone	4	2	410.5	2.7	0	6
**15**	EZEVUD	paracetamol	3	1	151.16	0.5	2	2
**16**	EZEWOY	doxorubicinone	8	3	414.4	1.6	5	9
**17,18**	VUKYUX/VUKYOR	1*R*-(-)-menthyl acetate	3	0	198.3	3.6	0	2

[a] The number of rotatable bonds, excluding terminal methyl groups.

[b] No XLogP3-AA data available for this compound.

Table 2: Calculated strain, conformational, and total relative energies of the observed molecular geometries and RMSD comparison of the guest with its optimised isolated conformer. Structures are numbered according to configuration. Multiple guests are labelled A to D.

MOF type		CSD Refcode	Config.[a]	Guest[b]	ΔE_{strain} (kJ/mol)	RMSD (Å)	ΔE_{conf} (kJ/mol)	ΔE_{total} (kJ/mol)	ΔE_{MOF} (kJ/mol)
ZnI_2	**1**	LABMOT		-	-	-	-	-	16.06
	**2**	XAZPEW	1		3.99	0.042	0.00	3.99	4.53
	**3**	XAZNUK	1	A	9.87	0.060	4.24	14.10	19.83
				B	3.31	0.071	0.01[c]	3.33	
				C	2.41	0.034	0.01[c]	2.42	
				D	7.81	0.124	0.01[c]	7.81	
	**4**	ZOQSUV	1	A	4.09	0.127	0.17	4.26	8.79
				B	3.37	0.129	0.16	3.53	
				C	6.73	0.101	0.18	6.90	
	**5**	XAZPAS	1		2.50	0.094	0.00	2.50	7.19
	**6**	CENBOP	1	A1	17.79	0.463	8.74	26.53	8.49
			2	A2	17.33	0.465	8.74	26.07	10.43
	**7**	GARLAQ	1		18.16	0.185	0.00	18.16	16.82
	**8**	LIRLIL	1	A	6.02	0.106	22.53	28.55	9.93
				B	23.47	0.577	5.14	28.61	
			2	A	6.75	0.102	21.78	28.53	9.98
				B	23.39	0.577	5.13	28.52	
	**9**	LIRLEH	1	A	19.93	0.423	3.81	23.76	9.10
				B	5.23	0.085	21.05	26.28	
			2	A	19.88	0.423	3.81	23.70	9.10
				B	5.74	0.099	21.03	26.77	
$ZnCl_2$	**10**	CENBUV	1		15.50	0.402	1.79	17.29	88.35
			2		14.86	0.405	1.80	16.65	89.21
			3		13.52	0.384	1.79	15.31	100.95
			4		13.31	0.371	1.82	15.13	101.83
	**11**	RECJAN	1		25.91	0.651	6.74	32.65	16.89
	**12**	EZEWUE	1	A1	11.06	0.387	0.56	11.62	14.25
			2	A1	12.63	0.415	0.56	13.19	17.06
			3	A2	10.99	0.385	0.57	11.56	14.26
			4	A2	12.94	0.420	0.56	13.50	16.84
	**13**	COQBIW	1	A	12.56	0.404	28.45	41.01	17.49
				B	16.24	0.481	19.98	36.22	
	**14**	EZEXAL	1	A1	8.38	0.298	6.85	15.23	9.30
			2	A2	8.26	0.299	6.85	15.11	9.21
			3	B1	18.13	0.943	5.03	23.16	17.05
			4	B2	18.20	0.940	5.02	23.22	16.99
			5	A	12.35	0.414	6.85	19.21	19.78
				B	18.43	0.971	5.03	23.46	
			6	A	12.46	0.432	6.82	19.28	18.07
				B	18.39	0.965	5.03	23.42	
	**15**	EZEVUD	1	A1	6.90	0.112	1.59	8.50	13.02
			2	A2	7.04	0.112	1.59	8.63	12.87
	**16**	EZEWOY	1	A1	41.19	0.620	79.84	121.04	13.17
			2	A2	40.80	0.601	79.75	120.55	13.28
	**17**	VUKYUX	1	A	4.66	0.195	0.45[c]	5.11	20.70
				B	4.61	0.143	0.02[c]	4.63	
$ZnBr_2$	**18**	VUKYOR	1	A	3.08	0.060	0.63[c]	3.71	3.41
				B	3.41	0.110	0.05[c]	3.46	

[a] For disordered structures, multiple configurations have been considered.

[b] Structures that contain a guest molecule that sits over a rotation axis/symmetry site have been labelled numerically, e.g. A1 and A2.

[c] Geometric comparisons show that the conformer in the experimental structure is the same as the global minimum energy conformer. This is therefore indicative of calculations errors due to convergence tolerances.

Table 3: Calculated strain, conformational, and total relative energies of the observed molecular geometries in the pure crystal structures studied, and RMSD comparison of the experimental mo- lecular geometry with its optimised isolated conformer. Structures are labelled with their associated CSD Refcodes. For crystal structures with Z'>1, the unique molecules are labelled alphabetically.

	CSD Refcode	Molecule	ΔE_{strain} (kJ/mol)	RMSD (Å)	ΔE_{conf} (kJ/mol)	ΔE_{total} (kJ/mol)
dapsone	DAPSUO05[15]		10.43	0.166	1.78	12.21
	DAPSUO15[16]		11.51	0.129	1.78	13.29
	DAPSUO18[17]	A	9.81	0.144	1.79	11.60
		B	10.71	0.171	1.83	12.54
		C	20.44	0.680	1.78	22.23
		D	12.11	0.139	0.83	12.94
nifedipine	BICCIZ02[18]	A	8.08	0.336	12.79	20.87
		B	13.42	0.288	12.71	26.13
	BICCIZ08[19]	A	20.52	0.903	12.94	33.46
		B	17.73	0.432	19.01	36.74
	BICCIZ09[19]	A	12.10	0.285	19.01	31.11
		B	14.66	0.301	19.00	33.66
	BICCIZ10[19]	A	9.51	0.175	19.00	28.50
		B	9.13	0.390	12.92	22.05
	BICCIZ12[19]		8.86	0.299	6.73	15.59
	BICCIZ14[19]		8.94	0.171	19.31	28.26
nifuroxazide	LEQTAC[20]		15.80	0.292	28.82	44.62
(+)-artemisinin	QNGHSU11[21]		2.47	0.062	0.04	2.51
	QNGHSU01[22]	A	2.15	0.035	0.02	2.17
		B	2.07	0.034	0.03	2.10
		C	2.18	0.035	0.01	2.19
		D	2.00	0.040	0.01	2.01
epiartemisinin	WIMMEK[23]		2.25	0.042	-	-
vanillin	YUHTEA01[24]	A	10.88	0.035	0.01	10.89
		B	12.44	0.065	0.01	12.45
		C	13.18	0.043	0.02	13.20
		D	15.70	0.054	0.01	15.72
paracetamol	HXACAN07[25]		8.49	0.072	1.76	10.25
	HXACAN08[25]		10.54	0.128	1.68	12.22
	HXACAN40[26]	A	13.47	0.138	1.76	15.23
		B	12.04	0.106	1.87	13.91
	HXACAN39[26]	A	13.74	0.137	1.62	15.36
		B	12.09	0.116	1.75	13.84
		C	13.59	0.140	1.61	15.20
		D	11.98	0.105	1.86	13.84
risperidone	WASTEP[27]		7.17	0.267	15.56	22.72
	WASTEP01[28]	A	9.61	0.206	15.55	25.16
		B	8.36	0.213	15.57	23.93
BBA-8,12-OMe	BOWHUU[10]	A	9.06	0.118	20.95	30.01
		B	23.99	0.200	3.80	27.78
BBA-8,10,12-OMe	2341232		28.27	0.598	5.02	33.29

Table 4: Calculated strain, conformational, and total relative energies of the observed molecular geometries in the cocrystal structures studied, and RMSD comparison of the experimental molecu- lar geometry with its optimised isolated conformer. Structures are labelled with their associated CSD Refcodes. Multiple configurations of structures are labelled numerically, and where multiple guests are present, they are labelled A to C.

	CSD Refcode	Config.	Molecule	ΔE_{strain} (kJ/mol)	RMSD (Å)	ΔE_{conf} (kJ/mol)	ΔE_{total} (kJ/mol)
trans-anethole	XOMLOA[29]			2.37	0.059	0.00	2.37
dapsone	ANSFON02[30]		A	11.10	0.205	0.06	11.16
			B	17.28	0.238	0.82	18.10
			C	15.97	0.165	0.02	15.99
	BIQNEW[31]			14.26	0.059	0.79	15.05
	BOPBEQ[32]		A	12.15	0.165	0.81	12.96
			B	14.19	0.079	0.06	14.25
	BOPBIU[32]		A	10.19	0.105	0.81	11.00
			B	14.41	0.066	0.00	14.41
	GAQYEE[33]	1	A	9.81	0.194	0.04	9.85
			B	13.40	0.269	0.08	13.48
		2	A	12.47	0.247	0.00	12.47
			B	10.99	0.178	0.06	11.05
	HADYUK[34]			16.85	0.136	1.84	18.70
	HADZAR[34]			15.23	0.087	0.81	16.04
	HASRUQ[35]		A	12.09	0.129	0.78	12.88
			B	14.33	0.185	0.00	14.33
	HASSAX[35]	1	A	15.11	0.209	0.03	15.14
		2	A	15.86	0.103	0.79	16.64
	HASSEB[35]	1	A	9.64	0.066	0.83	10.47
		2	A	9.65	0.070	0.82	10.47
	KIGNEV[36]			10.96	0.060	1.79	12.75
	KIGNIZ[36]			6.94	0.054	0.81	7.75
	LANYIK[37]			8.75	0.054	1.81	10.56
	NEBPOC[38]			10.70	0.079	0.05	10.75
	RUHDOP[39]		A	10.16	0.085	1.78	11.93
			B	11.93	0.400	0.79	12.72
	TIDZEN[40]		A	14.14	0.184	0.05	14.19
			B	12.01	0.098	0.83	12.84
	VOHKAG[41]			21.97	0.409	-0.01	21.96
	VOHKEK01[39]			10.73	0.131	0.78	11.50
	VOHKIO[41]			16.69	0.240	0.85	17.54
	VOHKOU[41]			17.75	0.171	0.88	18.63
	VOHKUA[41]			10.28	0.106	1.79	12.07
	YIPMIW[42]		A	10.92	0.146	0.81	11.73
			B	11.09	0.050	0.84	11.93
nifedipine	ASATOD[43]			8.54	0.202	19.02	27.56
	JENMOH[44]			14.99	0.242	19.03	34.02
	JENMUN[44]			12.90	0.551	12.71	25.61
	JENMUN01[44]			17.02	0.679	12.70	29.72
	QUPRUP[45]			10.54	0.236	19.00	29.54
	SUZGEA[46]			16.25	0.614	12.71	28.96
	WEKDUP[47]			9.12	0.232	19.10	28.22
nifuroxazide	RAVSIT[48]			16.10	0.199	28.86	44.96
	RAVSOZ[48]		A	13.60	0.230	11.28	24.88
			B	20.30	0.336	11.28	31.58
	RAWCAW[48]			15.49	0.285	28.86	44.35
(+)-artemisinin	FAWFOC[49]			6.14	0.026	0.01	6.15
	TALCOA[50]		A	7.63	0.169	0.03	7.66
			B	5.09	0.094	0.01	5.09

Table 4 continued from previous page

	CSD Refcode	Config.	Molecule	ΔE_{strain} (kJ/mol)	RMSD (Å)	ΔE_{conf} (kJ/mol)	ΔE_{total} (kJ/mol)
	TALCUG[50]			7.87	0.146	0.02	7.89
vanillin	DUMWEQ[51]			5.79	0.029	23.15	28.94
	FIVNEF[52]			4.26	0.025	23.14	27.39
	GIHCEH[53]			10.59	0.037	-0.01	10.58
	IWOFIL[54]	1	A	10.68	0.033	-0.01	10.68
			B	12.07	0.081	4.23	16.30
		2	A	10.71	0.034	-0.01	10.71
			B	12.03	0.079	4.23	16.26
	LEWSUD[55]		A	11.06	0.041	4.24	15.30
			B	5.44	0.031	23.14	28.58
	MAHKAJ[56]			10.28	0.082	-0.01	10.28
	MAHKAJ01[57]			10.29	0.081	-0.01	10.28
	MAHKEN[56]			18.71	0.033	-0.02	18.69
	NUTPOJ[58]			7.28	0.041	-0.01	7.27
	OBUBOE[59]			12.64	0.039	25.81	38.45
	PAMDIT[60]			12.69	0.054	-0.01	12.68
	ROFCEX[61]			12.20	0.050	-0.01	12.19
	UHAWAD[62]		A	14.63	0.046	-0.01	14.63
			B	14.11	0.057	0.00	14.11
	VESRIX[63]			8.04	0.073	-0.01	8.03
	VESYUQ[63]			16.51	0.038	-0.01	16.50
	YOLQOF[64]			17.40	0.065	-0.01	17.39
	YUYQAM[65]			9.92	0.084	25.81	35.73
	PEDMUK[66]			10.73	0.056	23.14	33.87
	WEGFUN[67]			8.72	0.036	4.24	12.95
paracetamol	AHEPUY[68]	1		13.42	0.117	0.38	13.80
		2		14.82	0.079	0.37	15.19
	AMUBAM[69]		A	6.83	0.088	0.38	7.20
			B	7.15	0.038	0.31	7.46
	ARAFOR[70]			9.38	0.068	1.68	11.06
	BODSUL[71]			8.60	0.083	1.65	10.25
	COKCEL[72]			19.03	0.134	0.44	19.47
	HUMJEE[73]			10.75	0.126	0.44	11.19
	JEMTEE[74]		A	7.78	0.141	1.63	9.41
			B	7.07	0.117	1.89	8.96
	KETYUF[75]		A	10.20	0.084	0.37	10.62
			B	10.77	0.135	0.36	11.13
	KETZAM[75]			13.89	0.078	0.36	14.25
	KETZEQ[75]			12.54	0.077	0.39	12.93
	KETZIU[75]	1		12.18	0.086	0.43	12.61
		2		12.27	0.083	0.32	12.59
	KIGLUI01[76]			8.57	0.186	1.77	10.34
	KUNTUK06[77]		A	17.09	0.187	1.73	18.82
			B	8.44	0.059	1.60	10.05
	LUJSIT[76]			14.22	0.134	1.81	16.03
	LUJSOZ[76]			5.17	0.033	0.54	5.71
	MUPPES01[78]			9.30	0.130	0.38	9.68
	MUPPES02[78]		A	11.18	0.194	0.38	11.56
			B	10.71	0.149	0.37	11.08
	MUPPIW[79]			10.79	0.074	1.67	12.47
	MUPPUI[79]			10.55	0.053	1.60	12.16
	MUPQAP[79]		A	11.15	0.129	1.60	12.75
			B	8.62	0.119	1.76	10.38
	MUPQET[79]		A	6.32	0.107	1.65	7.97
			B	17.72	0.036	1.69	19.41
	NIDPIB[80]			13.00	0.139	0.51	13.50

Table 4 continued from previous page

CSD Refcode	Config.	Molecule	ΔE_{strain} (kJ/mol)	RMSD (Å)	ΔE_{conf} (kJ/mol)	ΔE_{total} (kJ/mol)
OMISIM[81]			8.68	0.023	0.39	9.07
OMISIM01[82]		A	8.77	0.057	0.42	9.19
		B	8.92	0.072	0.42	9.34
		C	8.02	0.022	0.40	8.42
OMISIM02[82]		A	8.76	0.055	0.43	9.19
		B	8.93	0.070	0.42	9.35
		C	8.11	0.021	0.32	8.32
RECJOC[83]			6.35	0.034	0.54	6.89
WAFNAT[84]			10.95	0.060	0.55	11.49
WIGBUL[85]	1	A	9.95	0.089	0.43	10.37
		B	10.08	0.103	0.36	10.43
	2	A	12.89	0.070	0.42	13.31
		B	11.61	0.075	0.31	11.92
WIGCAS[85]		A	10.43	0.057	0.35	10.78
		B	11.44	0.127	0.37	11.81
WIGCEW[85]			15.47	0.136	0.37	15.84
XOMWOL[86]			12.74	0.152	1.84	14.58

Table 5: Framework strain. Where multiple configurations of the structure were considered, these are labelled numerically.

MOF type		CSD Refcode	Space Group	SG used for optimisation	Config.	ΔE_{MOF} (kJ/mol)	ΔE_{MOF} per 81 atoms (kJ/mol)	Volume dev (%)
ZnI_2	**1**	LABMOT	*C2/c*	*P*1		64.24	16.06	0.62
	~~**2**~~	~~XAZPEW~~	~~*C2*~~	~~*C2*~~		~~18.13~~	~~4.53~~	~~-0.60~~
	**3**	XAZNUK	*P*-1	*P*-1		79.33	19.83	0.98
	**4**	ZOQSUV	*C2/c*	*C2/c*		35.16	8.79	-1.66
	**5**	XAZPAS	*C2/c*	*C2/c*		28.77	7.19	-1.64
	**6**	CENBOP	*C2/c*	*Cc*	1	33.96	8.49	-0.73
					2	41.73	10.43	-0.62
	**7**	GARLAQ	*C2/c*	*C2/c*		67.29	16.82	-1.01
	**8**	LIRLIL	*C2/c*	*Cc*	1	39.74	9.93	-0.83
					2	39.90	9.98	-1.17
	**9**	LIRLEH	*P2/n*	*Pc*	1	72.83	9.10	-0.28
					2	72.83	9.10	-0.28
$ZnCl_2$	**10**	CENBUV	*C2/c*	*P*1	1	353.38	88.35	-15.60
					2	356.86	89.21	-15.22
					3	403.80	100.95	-15.51
					4	407.31	101.83	-15.76
	**11**	RECJAN	*P21/c*	*P21/c*		135.08	16.89	-4.96
	**12**	EZEWUE	*C2/c*	*P*1	1	56.99	14.25	2.70
					2	68.23	17.06	3.11
					3	57.05	14.26	2.67
					4	67.36	16.84	3.05
	**13**	COQBIW	*C2*	*C2*		69.94	17.49	-0.39
	**14**	EZEXAL	*C2/c*	*Cc*	1	37.18	9.30	0.05
					2	36.86	9.21	0.04
					3	68.19	17.05	-1.29
					4	67.95	16.99	-1.29
					5	79.13	19.78	-2.36
					6	72.28	18.07	-1.31
	**15**	EZEVUD	*C2/c*	*Cc*	1	52.08	13.02	0.33
					2	51.50	12.87	0.27
	**16**	EZEWOY	*C2/c*	*Cc*	1	52.68	13.17	2.21
					2	53.12	13.28	2.15
	**17**	VUKYUX	*C2*	*C2*		82.82	20.70	-6.27
$ZnBr_2$	**18**	VUKYOR	*C2*	*C2*		13.65	3.41	0.05

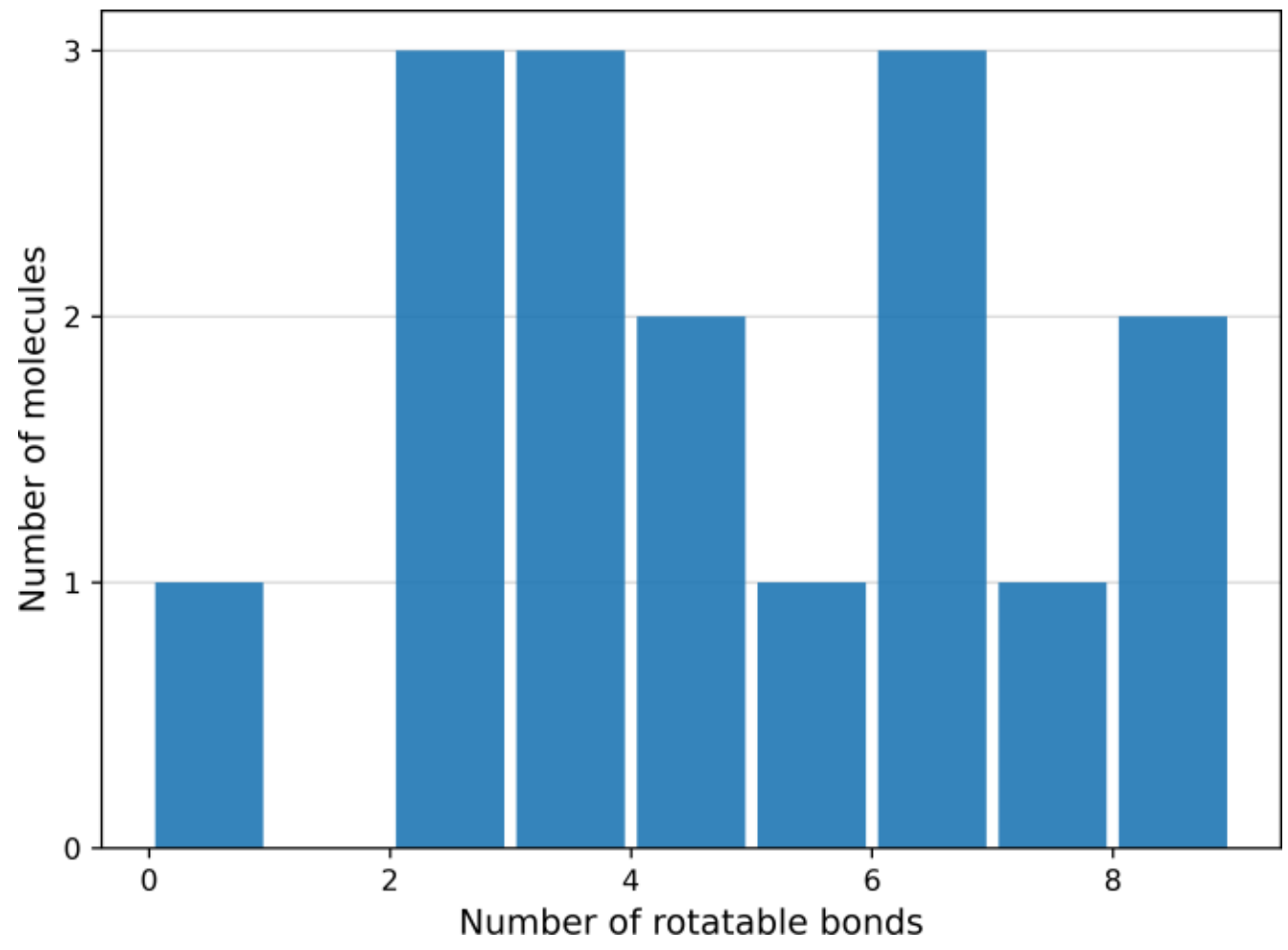

Figure 2: Histogram detailing the distribution of the number of intramolecular rotatable bonds of the 17 CS guest molecules studied.

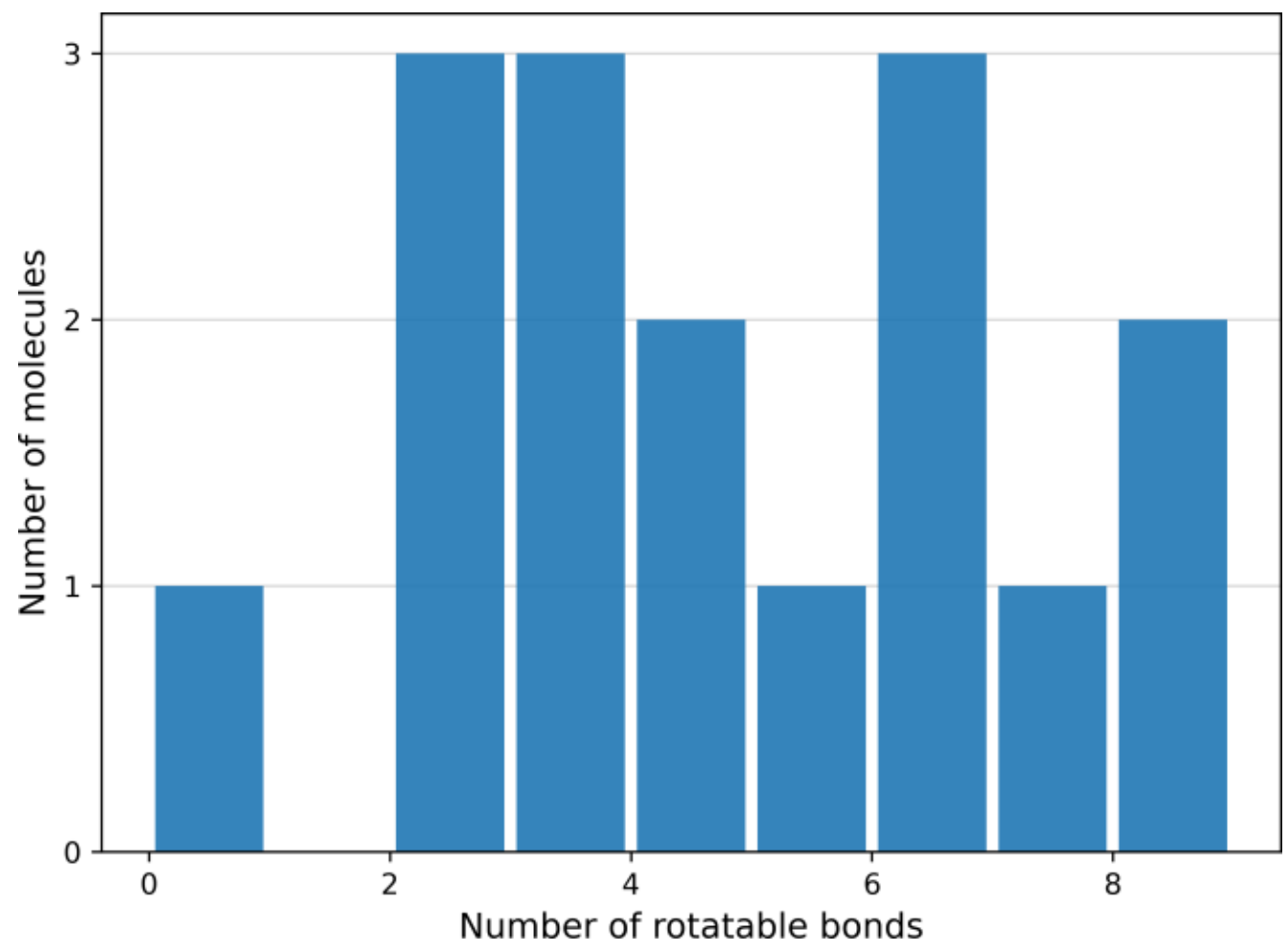

Figure 3: Histogram detailing the distribution of the total relative energy of molecules in cocrystals, broken down by the molecule of interest (comparable to CS structures) and detailed in the figure legend.

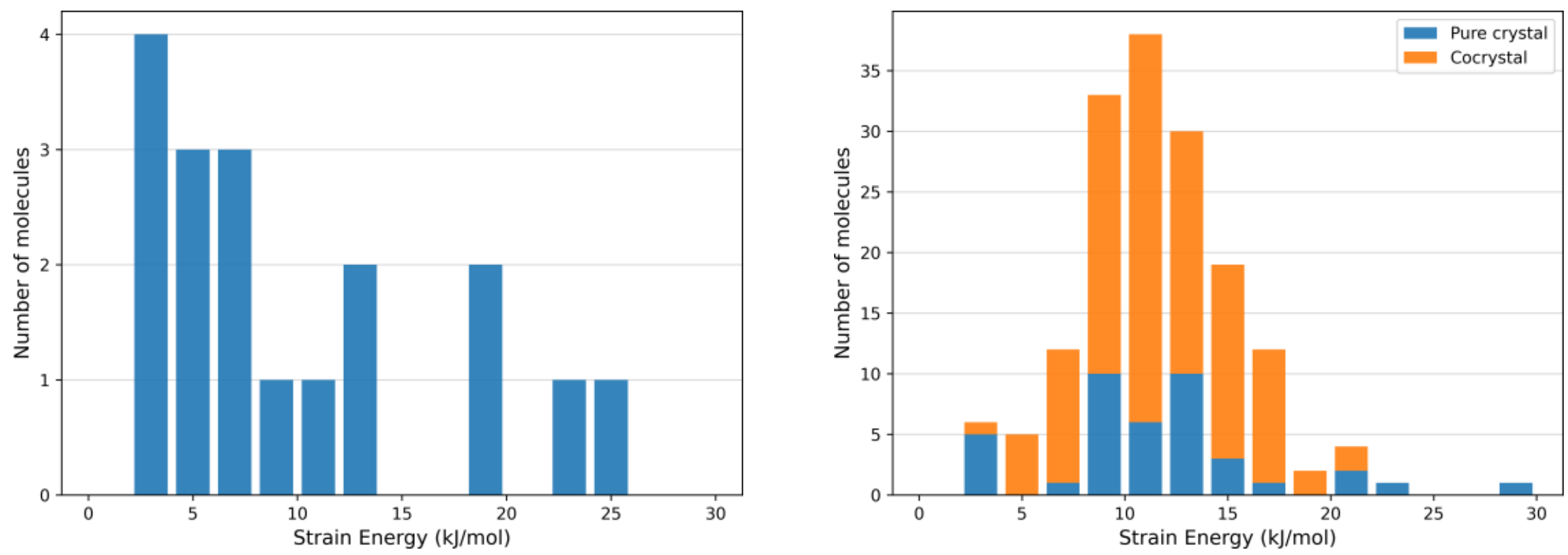

(a) (b)

Figure 4: Histogram detailing the distribution of intramolecular strain energies ΔE_{strain} of (a) the CS guest molecules which have pure and/or cocrystal structures, and (b) of those guests molecules in their pure and/or cocrystal structures

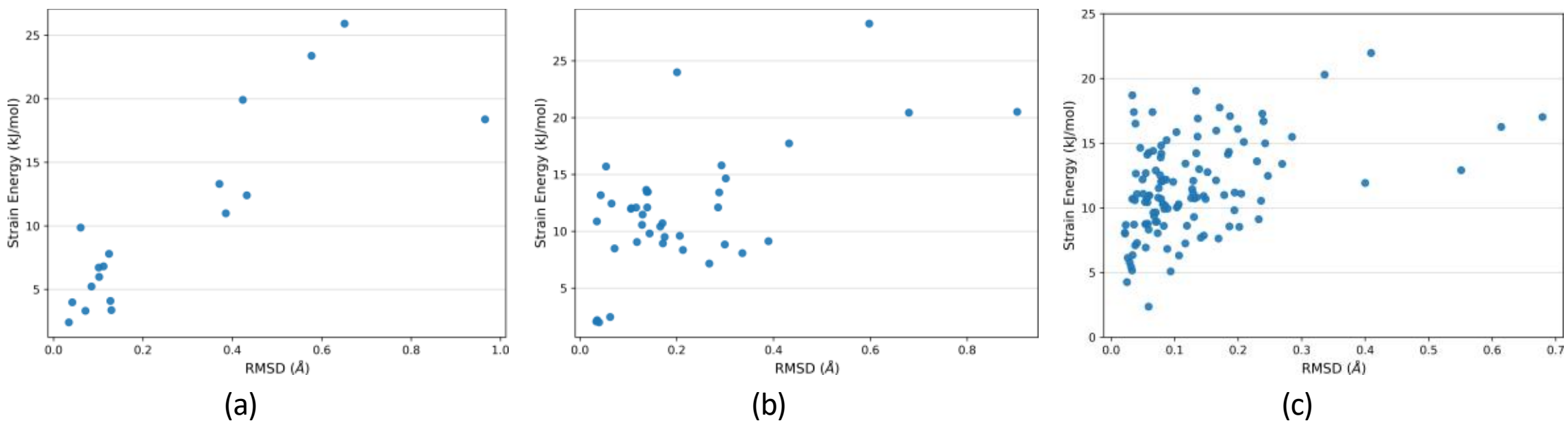

Figure 5: Plot of ΔE_{strain} against RMSD of atomic positions between the optimised crystalline molecular geometry and the conformation of the optimised isolated molecule, for (a) CS guests with pure and/or cocrystal structures, (b) molecules in pure crystal structures, and (c) molecules in cocrystal structures.

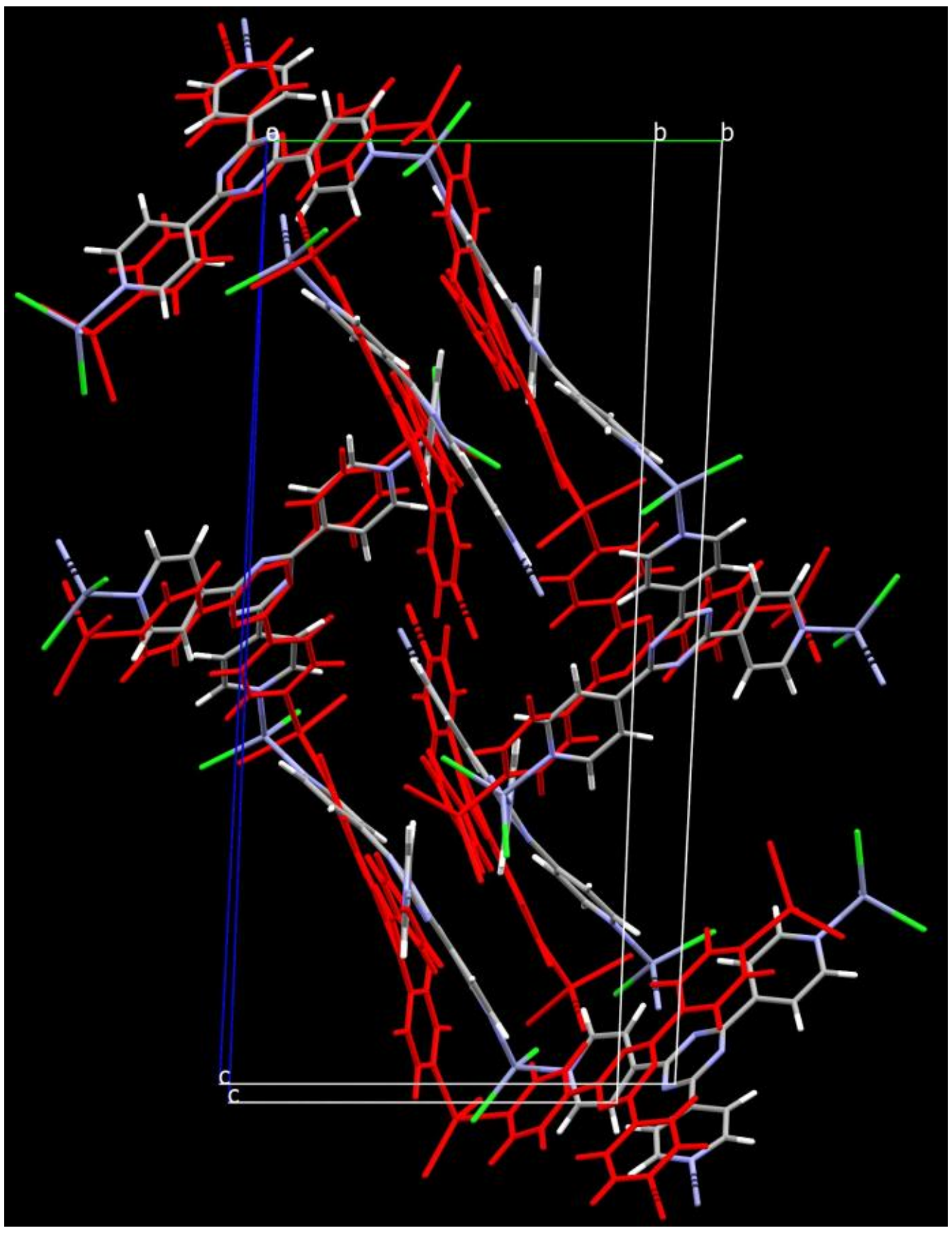

Figure 6: Overlay comparison of the empty framework in structure 10 (CENBUV) before (element colours) and after (red) framework re-optimisation for calculation of framework strain energies. Perspective view down the *a* axis.

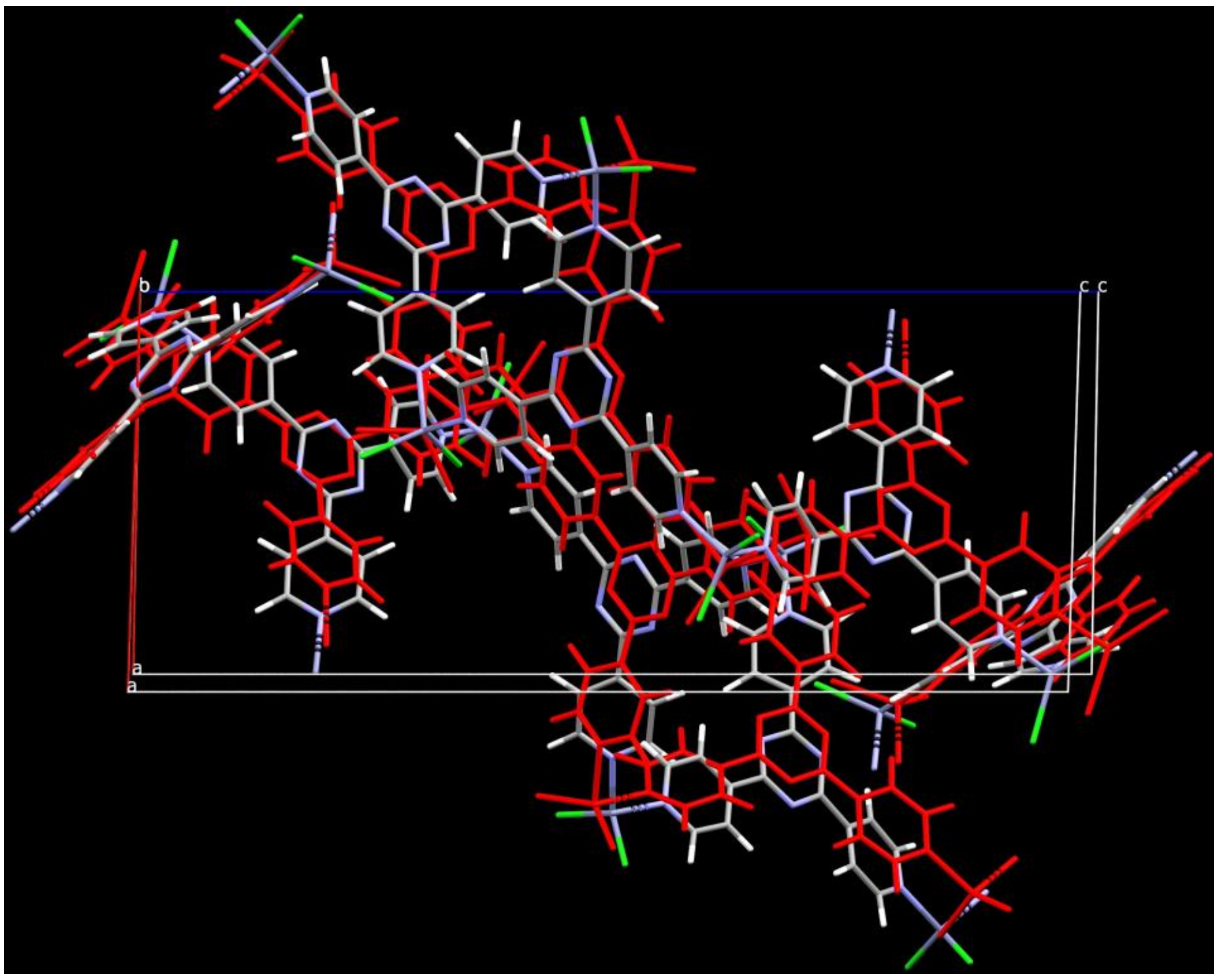

Figure 7: Overlay comparison of the empty framework in structure 10 (CENBUV) before (element colours) and after (red) framework re-optimisation for calculation of framework strain energies. Perspective view down the *b* axis.

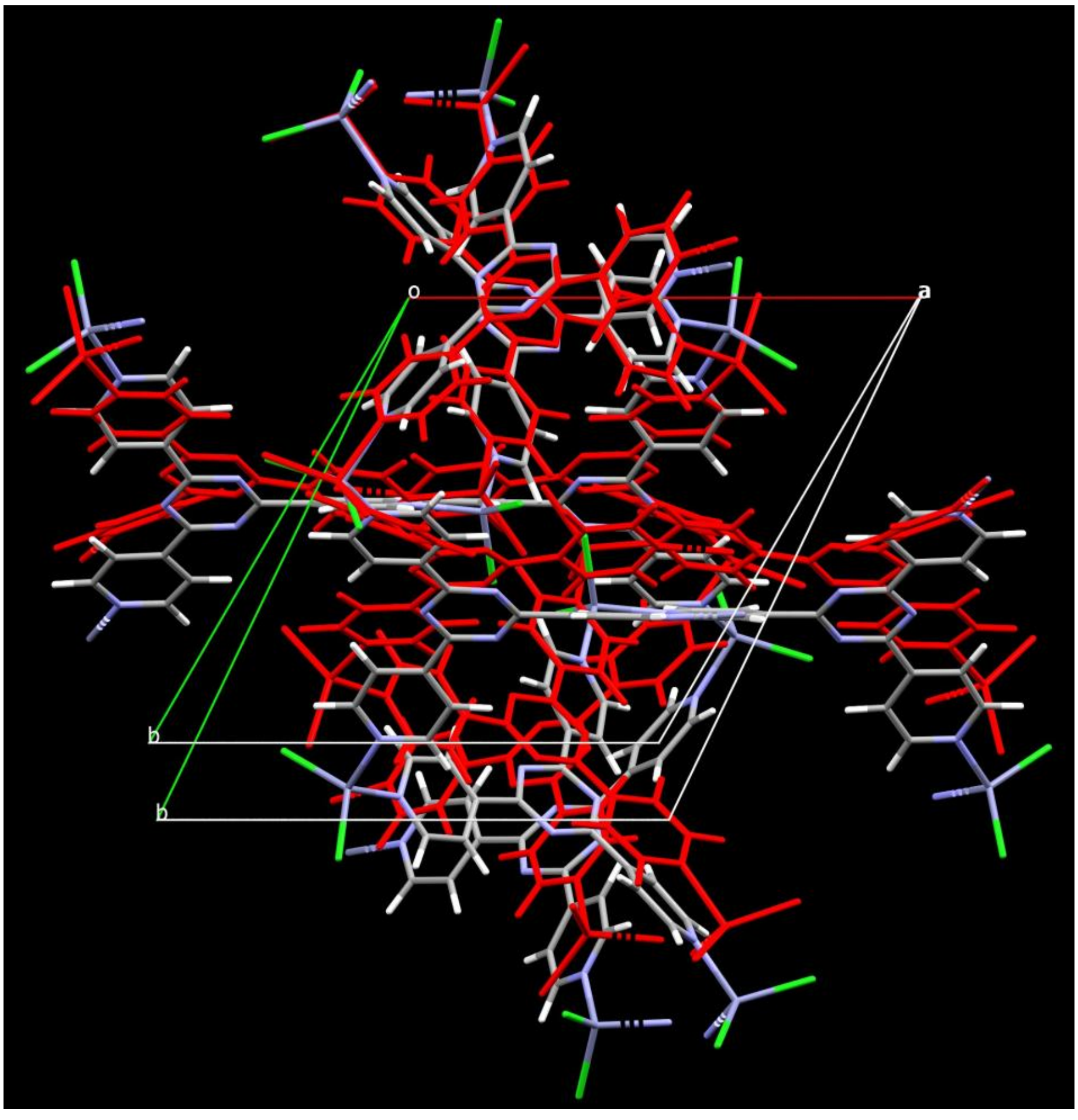

Figure 8: Overlay comparison of the empty framework in structure 10 (CENBUV) before (element colours) and after (red) framework re-optimisation for calculation of framework strain energies. Perspective view down the *c* axis.

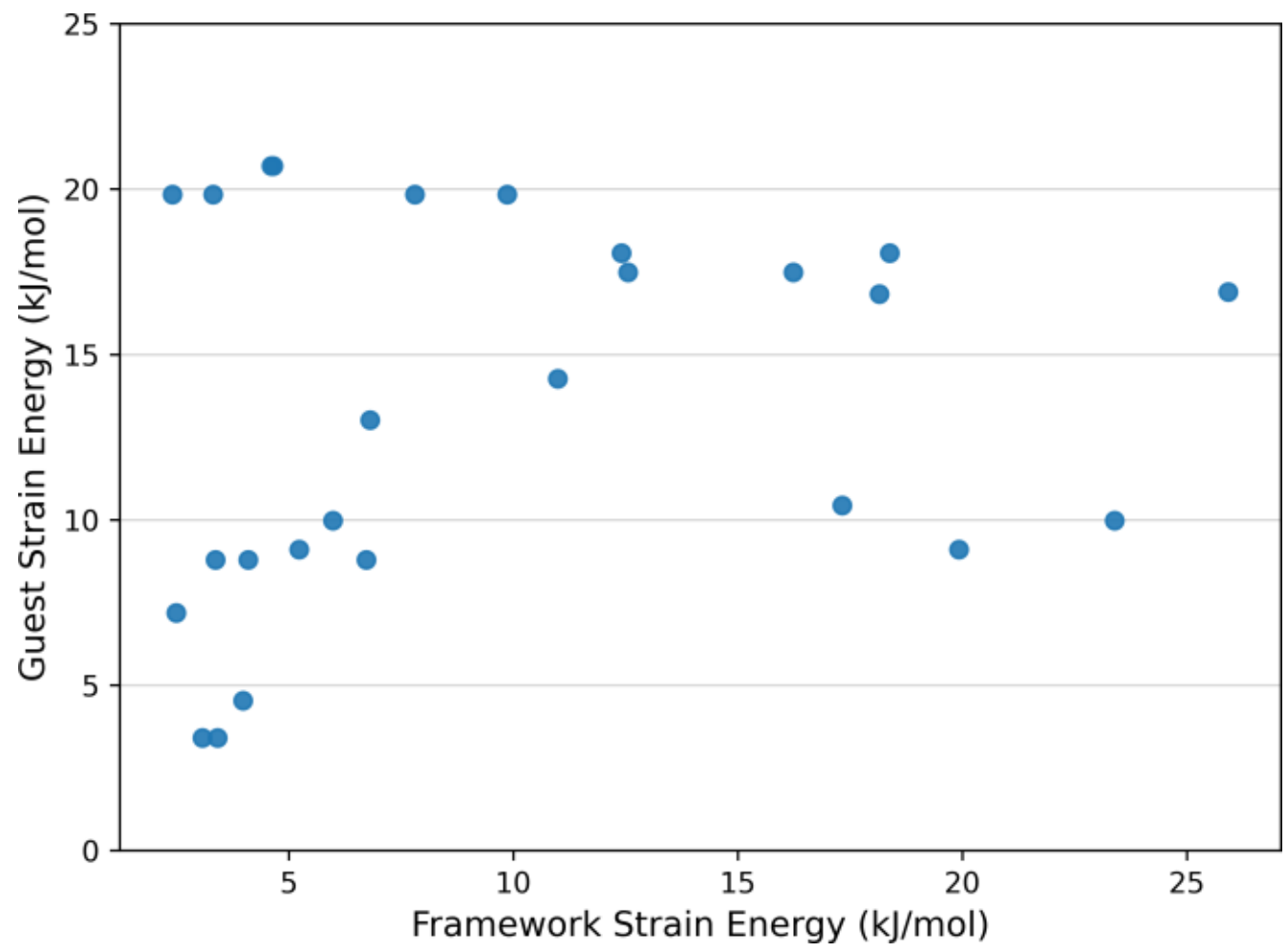

Figure 9: Plot of ΔE_{strain} of guest molecules against ΔE_{MOF}.

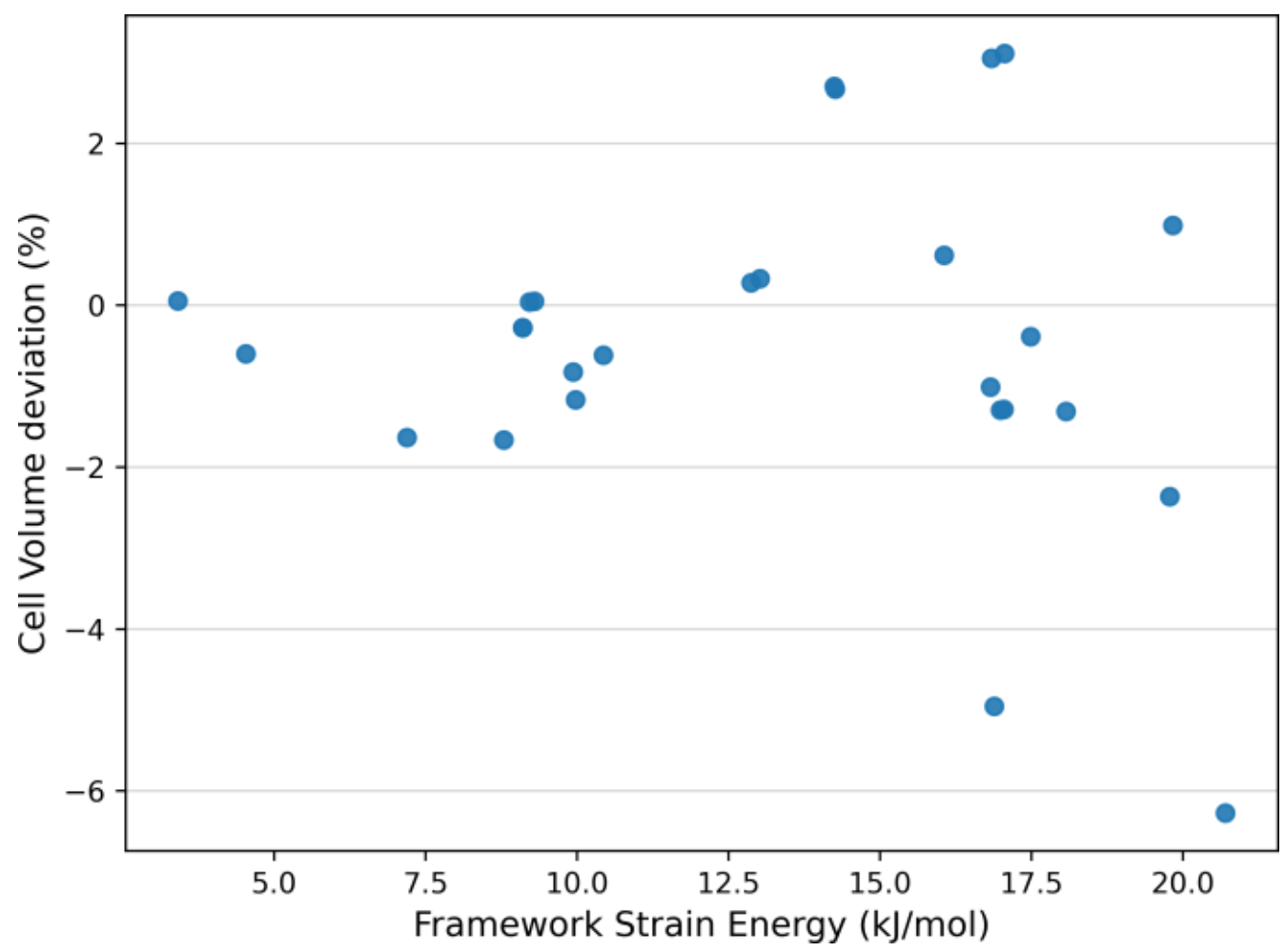

Figure 10: Plot of ΔE_{MOF} of the CS framework with all guest and solvent molecules are removed versus percentage change in cell volume.

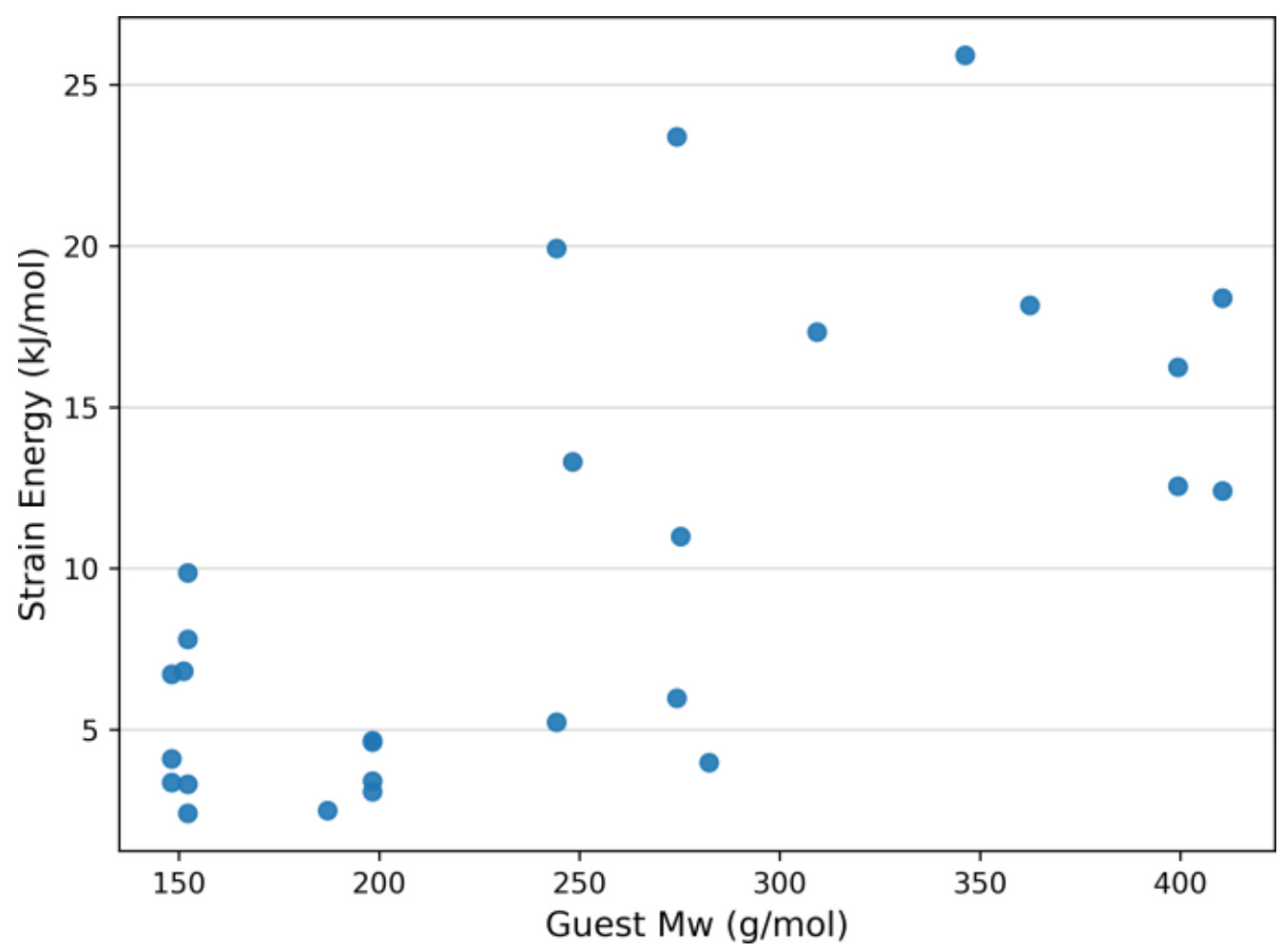

Figure 11: ΔE_{strain} versus the molecular weight of the CS guest molecules.

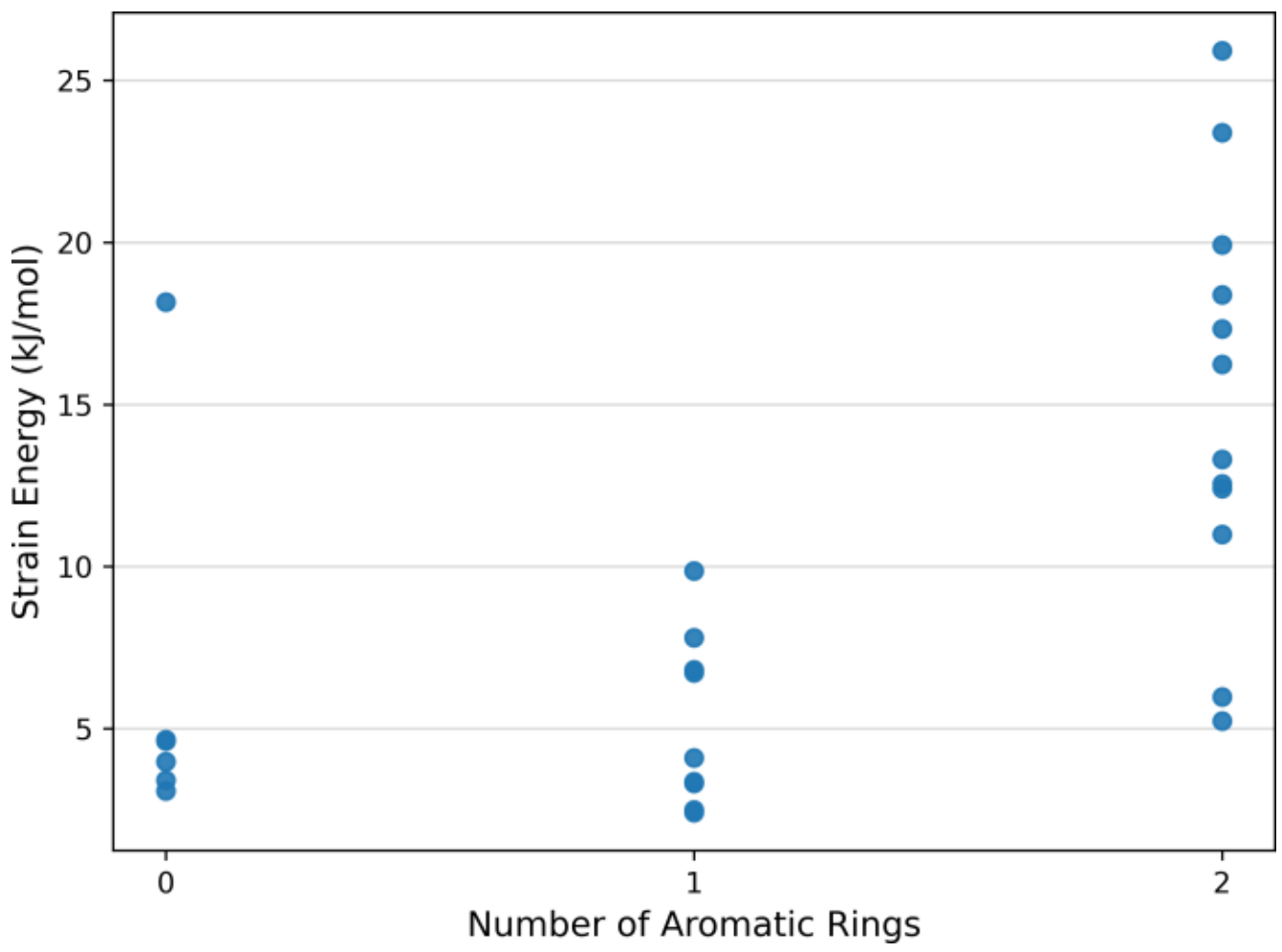

Figure 12: ΔE_{strain} versus the number of aromatic rings in the chosen CS guest molecules.

References

(1) Dovesi, R.; Erba, A.; Orlando, R.; Zicovich-Wilson, C. M.; Civalleri, B.; Maschio, L.; Rérat, M.; Casassa, S.; Baima, J.; Salustro, S.; Kirtman, B. *WIREs Computational Molecular Sci- ence* **2018**, *8*, e1360.

(2) Laun, J.; Oliveira, D. V.; Bredow, T. *Journal of Computational Chemistry* **2018**, *39*, 1285–1290.

(3) Peintinger, M. F.; Oliveira, D. V.; Bredow, T. *Journal of Computational Chemistry* **2013**, *34*, 451–459.

(4) Civalleri, B.; Zicovich-Wilson, C. M.; Valenzano, L.; Ugliengo, P. *CrystEngComm* **2008**, *10*, 405–410.

(5) Grimme, S.; Antony, J.; Ehrlich, S.; Krieg, H. *The Journal of Chemical Physics* **2010**, *132*, 154104.

(6) Brandenburg, J. G.; Alessio, M.; Civalleri, B.; Peintinger, M. F.; Bredow, T.; Grimme, S. *The Journal of Physical Chemistry A* **2013**, *117*, 9282–9292.

(7) Kruse, H.; Grimme, S. *The Journal of Chemical Physics* **2012**, *136*, 154101.

(8) Pracht, P.; Bohle, F.; Grimme, S. *Physical Chemistry Chemical Physics* **2020**, *22*, 7169– 7192.

(9) Bruno, I. J.; Cole, J. C.; Kessler, M.; Luo, J.; Motherwell, W. D. S.; Purkis, L. H.; Smith, B. R.; Taylor, R.; Cooper, R. I.; Harris, S. E.; Orpen, A. G. *Journal of Chemical Information and Computer Sciences* **2004**, *44*, 2133–2144.

(10) Carroll, R. C.; Coles, S. J. *IUCrJ* **2024**, *11*, 578–586.

(11) Hoshino, M.; Khutia, A.; Xing, H.; Inokuma, Y.; Fujita, M. *IUCrJ* **2016**, *3*, 139–151.

(12) Ramadhar, T. R.; Zheng, S.-L.; Chen, Y.-S.; Clardy, J. *Chemical Communications* **2015**, *51*, 11252–11255.

(13) Ramadhar, T. R.; Zheng, S.-L.; Chen, Y.-S.; Clardy, J. *Acta Crystallographica Section A Foundations and Advances* **2015**, *71*, 46–58.

(14) Spek, A. L. *Acta Crystallographica Section C* **2015**, *71*, 9–18.

(15) Bertolasi, V.; Ferretti, V.; Gilli, P.; De Benedetti, P. G. *J. Chem. Soc., Perkin Trans. 2* **1993**, 213–219.

(16) Braun, D. E.; Krüger, H.; Kahlenberg, V.; Griesser, U. J. *Crystal Growth & Design* **2017**, *17*, 5054–5060.

(17) Braun, D. E.; Vickers, M.; Griesser, U. J. *Molecular Pharmaceutics* **2019**, *16*, 3221–3236.

(18) Gunn, E.; Guzei, I. A.; Cai, T.; Yu, L. *Crystal Growth & Design* **2012**, *12*, 2037–2043.

(19) Gui, Y.; Yao, X.; Guzei, I. A.; Aristov, M. M.; Yu, J.; Yu, L. *Chemistry of Materials* **2020**, *32*, 7754–7765.

(20) Pniewska, B.; Januchowski, M. *Polish Journal of Chemistry* **1998**, *72*, 2629.

(21) Yadav, J.; Thirupathaiah, B.; Srihari, P. *Tetrahedron* **2010**, *66*, 2005–2009.

(22) Chan, K.-L.; Yuen, K.-H.; Takayanagi, H.; Janadasa, S.; Peh, K.-K. *Phytochemistry* **1997**, *46*, 1209–1214.

(23) Jefford, C. W.; Burger, U.; Millasson-Schmidt, P.; Bernardinelli, G.; Robinson, B. L.; Peters, W. *Helvetica Chimica Acta* **2000**, *83*, 1239–1246.

(24) Nieger, M. *CSD Communication* **2007**, DOI: https://doi.org/10.5517/ccq45ww.

(25) Nichols, G.; Frampton, C. S. *Journal of Pharmaceutical Sciences* **1998**, *87*, 684–693.

(26) Reiss, C. A.; Mechelen, J. B. v.; Goubitz, K.; Peschar, R. *Acta Crystallographica Section C* **2018**, *74*, 392–399.

(27) Peeters, O. M.; Blaton, N. M.; De Ranter, C. J. *Acta Crystallographica Section C* **1993**, *49*, 1698–1700.

(28) Wang, D.-H.; Zhou, M.-H.; Hu, X.-R. *Acta Crystallographica Section E* **2006**, *62*, o3527– o3528.

(29) Guo, F.; Guo, W. S.; Toda, F. *CrystEngComm* **2003**, *5*, 45–47.

(30) Yathirajan, H. S.; Nagaraja, P.; Nagaraja, B.; Bhaskar, B. L.; Lynch, D. E. *CSD Commu- nication* **2007**, DOI: 10.5517/cc8l7bd.

(31) Smith, G.; Wermuth, U. D. *Chemical Crystallography* **2013**, *43*, 664–670.

(32) Braun, D. E.; Gelbrich, T.; Griesser, U. J. *CrystEngComm* **2019**, *21*, 5533–5545.

(33) Smith, G.; Wermuth, U. D. *Acta Crystallographica Section E* **2012**, *68*, o494.

(34) Zhao, C.; Li, W.; Li, Z.; Hu, W.; Zhang, S.; Wu, S. *CrystEngComm* **2021**, *23*, 6690–6702.

(35) Lemmer, H.; Stieger, N.; Liebenberg, W.; Caira, M. R. *Crystal Growth & Design* **2012**, *12*, 1683–1692.

(36) Martins, I.; Martins, M.; Fernandes, A.; André, V.; Duarte, M. T. *CrystEngComm* **2013**, *15*, 8173–8179.

(37) Smith, G.; Wermuth, U. D. *Acta Crystallographica Section E* **2012**, *68*, o669.

(38) *Journal of Molecular Structure* **2017**, *1149*, 452–456.

(39) He, H.; Jiang, L.; Zhang, Q.; Huang, Y.; Wang, J.-R.; Mei, X. *CrystEngComm* **2015**, *17*, 6566–6574.

(40) Babashkina, M. G.; Safin, D. A.; Robeyns, K.; Brzuszkiewicz, A.; Kozlowski, H.; Garcia, Y. *CrystEngComm* **2012**, *14*, 1324–1329.

(41) Jiang, L.; Huang, Y.; Zhang, Q.; He, H.; Xu, Y.; Mei, X. *Crystal Growth & Design* **2014**, *14*, 4562–4573.

(42) Chappa, P.; Maruthapillai, A.; Voguri, R.; Dey, A.; Ghosal, S.; Basha, M. A. *Crystal Growth & Design* **2018**, *18*, 7590–7598.

(43) Caira, M. R.; Robbertse, Y.; Bergh, J. J.; Song, M.; De Villiers, M. M. *Journal of Pharma- ceutical Sciences* **2003**, *92*, 2519–2533.

(44) Yu, Q.; Yan, Z.; Bao, J.; Wang, J.-R.; Mei, X. *Chem. Commun.* **2017**, *53*, 12266–12269.

(45) Klimakow, M.; Rademann, K.; Emmerling, F. *Crystal Growth & Design* **2010**, *10*, 2693– 2698.

(46) Schultheiss, N.; Roe, M.; Smit, J. P. *Acta Crystallographica Section E* **2010**, *66*, o2297– o2298.

(47) Batzdorf, L.; Zientek, N.; Rump, D.; Fischer, F.; Maiwald, M.; Emmerling, F. *Journal of Molecular Structure* **2017**, *1133*, 18–23.

(48) Covaci, O.-I.; Mitran, R.-A.; Buhalteanu, L.; Dumitrescu, D. G.; Shova, S.; Manta, C.-M. *CrystEngComm* **2017**, *19*, 3584–3591.

(49) Makadia, J.; Madu, S. J.; Arroo, R.; Seaton, C. C.; Li, M. *CrystEngComm* **2022**, *24*, 1056– 1067.

(50) Karki, S.; Friščić, T.; Fábián, L.; Jones, W. *CrystEngComm* **2010**, *12*, 4038–4041.

(51) Parvathy, G.; Kaliammal, R.; Sankaranarayanan, K.; Arivanandhan, M.; Krishna Kumar, M.; Sudhahar, S. *Journal of Molecular Structure* **2020**, *1217*, 128406.

(52) Halim, S. N. A.; Nadzri, I. *CSD Communication* **2014**, DOI: 10.5517/cc11wdnz.

(53) Suresh, K.; Goud, N. R.; Nangia, A. *Chemistry – An Asian Journal*, *8*, 3032–3041.

(54) Sudharsana, N.; Krishnakumar, V.; Nagalakshmi, R. *Eur. Phys. J. Plus* **2016**, *131*, 348.

(55) Alhalaweh, A.; George, S.; Basavoju, S.; Childs, S. L.; Rizvi, S. A. A.; Velaga, S. P. *Crys- tEngComm* **2012**, *14*, 5078–5088.

(56) Braga, D.; Grepioni, F.; Maini, L.; Mazzeo, P. P.; Rubini, K. *Thermochimica Acta* **2010**, *507-508*, 1–8.

(57) Lee, T.; Chen, H. R.; Lin, H. Y.; Lee, H. L. *Crystal Growth & Design* **2012**, *12*, 5897–5907.

(58) Childs, S. L.; Kandi, P.; Lingireddy, S. R. *Molecular Pharmaceutics* **2013**, *10*, 3112–3127.

(59) How, F. N.-F.; Amalina, M. S.; Khaledi, H.; Mohd Ali, H. *Acta Crystallographica Section E* **2011**, *67*, o3168.

(60) Peraka, K. S.; Lusi, M.; Bajpai, A.; Zaworotko, M. J. *Crystal Growth & Design* **2017**, *17*, 959–962.

(61) Nemec, V.; Fotović, L.; Vitasović, T.; Cinčić, D. *CrystEngComm* **2019**, *21*, 3251–3255.

(62) Krishna, G. R.; Shi, L.; Bag, P. P.; Sun, C. C.; Reddy, C. M. *Crystal Growth & Design* **2015**, *15*, 1827–1832.

(63) Amombo Noa, F. M.; Mehlana, G. *CrystEngComm* **2018**, *20*, 896–905.

(64) Usman, A.; Chantrapromma, S.; Fun, H.-K.; Poh, B.-L.; Karalai, C. *Acta Crystallographica Section C* **2002**, *58*, o48–o50.

(65) Thipparaboina, R.; Kumar, D.; Mittapalli, S.; Balasubramanian, S.; Nangia, A.; Shastri, N. R. *Crystal Growth & Design* **2015**, *15*, 5816–5826.

(66) Pandit, D.; Chadha, R.; Laha, B.; Gautam, M. K.; Karan, M.; Mandal, S. K. *Crystal Growth & Design* **2022**, *22*, 2218–2229.

(67) Yang, S.-Y.; Zhao, F.-K.; Pang, H.; Chen, L.-Z.; Shi, R.-B.; Fang, B.-H. *Journal of Molecular Structure* **2022**, *1265*, 133335.

(68) Oswald, I. D. H.; Motherwell, W. D. S.; Parsons, S.; Pulham, C. R. *Acta Crystallographica Section E* **2002**, *58*, o1290–o1292.

(69) Elbagerma, M. A.; Edwards, H. G. M.; Munshi, T.; Scowen, I. J. *CrystEngComm* **2011**, *13*, 1877–1884.

(70) Al-Ani, A. J.; Sugden, P.; Wilson, C. C.; Castro-Dominguez, B. *Crystal Growth & Design* **2021**, *21*, 3310–3315.

(71) Manonmani, M.; Balakrishnan, C.; Ahamed, S. R.; Vinitha, G.; Meenakshisundaram, S.; Sockalingam, R. *Journal of Molecular Structure* **2019**, *1190*, 1–10.

(72) Oswald, I. D. H.; Pulham, C. R. *CrystEngComm* **2008**, *10*, 1114–1116.

(73) Parkin, A.; Parsons, S.; Pulham, C. R. *Acta Crystallographica Section E* **2002**, *58*, o1345– o1347.

(74) Suzuki, N.; Kawahata, M.; Yamaguchi, K.; Suzuki, T.; Tomono, K.; Fukami, T. *Drug De- velopment and Industrial Pharmacy* **2018**, *44*, 582–589.

(75) Sander, J. R. G.; Bučar, D.-K.; Henry, R. F.; Giangiorgi, B. N.; Zhang, G. G. Z.; MacGilli- vray, L. R. *CrystEngComm* **2013**, *15*, 4816–4822.

(76) Karki, S.; Friščić, T.; Fábián, L.; Laity, P. R.; Day, G. M.; Jones, W. *Advanced Materials* **2009**, *21*, 3905–3909.

(77) Zakharov, B. A.; Ogienko, A. G.; Yunoshev, A. S.; Ancharov, A. I.; Boldyreva, E. V. *Crys- tEngComm* **2015**, *17*, 7543–7550.

(78) Vrcelj, R. M.; Clark, N. I.; Kennedy, A. R.; Sheen, D. B.; Shepherd, E. E.; Sherwood, J. N. *Journal of Pharmaceutical Sciences* **2003**, *92*, 2069–2073.

(79) Oswald, I. D. H.; Allan, D. R.; McGregor, P. A.; Motherwell, W. D. S.; Parsons, S.; Pulham, C. R. *Acta Crystallographica Section B* **2002**, *58*, 1057–1066.

(80) André, V.; M. da Piedade, M. F.; Duarte, M. T. *CrystEngComm* **2012**, *14*, 5005–5014.

(81) Fabbiani, F. P. A.; Allan, D. R.; Dawson, A.; David, W. I. F.; McGregor, P. A.; Oswald, I. D. H.; Parsons, S.; Pulham, C. R. *Chem. Commun.* **2003**, 3004–3005.

(82) Ward, M. R.; Oswald, I. D. H. *CrystEngComm* **2019**, *21*, 4437–4443.

(83) Andrusenko, I.; Hitchen, J.; Mugnaioli, E.; Potticary, J.; Hall, S. R.; Gemmi, M. *Symmetry* **2022**, *14*, DOI: 10.3390/sym14030431.

(84) Fabbiani, F. P. A.; Allan, D. R.; David, W. I. F.; Moggach, S. A.; Parsons, S.; Pulham, C. R. *CrystEngComm* **2004**, *6*, 505–511.

(85) Srirambhatla, V. K.; Kraft, A.; Watt, S.; Powell, A. V. *Crystal Growth & Design* **2012**, *12*, 4870–4879.

(86) McGregor, P. A.; Allan, D. R.; Parsons, S.; Pulham, C. R. *Journal of Pharmaceutical Sci- ences* **2002**, *91*, 1308–1311.
